# Supplementary material for: Spatial analysis of unimproved drinking water source in East Africa: Using Demographic and Health Survey (DHS) data from 2012–2023
Source: PLoS One. 2025 Mar 20;20(3):e0318189. doi: 10.1371/journal.pone.0318189 (PMC11925304; doi:10.1371/journal.pone.0318189)
Supplement: Attachment — Submitted filename: Reviewer Evaluation results No-PONE-D-24-33669with 15 pages.pdf [file pone.0318189.s004.pdf]

TITLE: Spatial analysis of unimproved drinking water source in East Africa: Based on demographic and health survey data, 2012-2022Multinomial Logistic Regression

Manuscript #: PONE-D-24-33669

1. GENERAL COMMENTS:

The manuscript titled "Spatial Analysis of Unimproved Drinking Water Sources in East Africa: Based on Demographic and Health Survey Data, 2012-2022" tackles an important public health issue with substantial implications for East Africa, where access to safe drinking water remains limited. The topic is timely and relevant, given the health risks associated with unimproved water sources.

The authors utilize demographic and health survey data from 12 East African countries, providing a strong analytical base. The application of spatial methods, such as Global Moran's I and spatial scan statistics, adds rigor to the findings. Identifying significant spatial clusters of unimproved drinking water sources is a valuable contribution that can help in effectively targeting interventions.

The recommendations in the conclusion are practical, emphasizing stakeholder collaboration, including governments and international organizations, to improve water quality and ensure sustainable access to safe drinking water in East Africa.

However, several improvements could enhance the manuscript. The background section would benefit from additional context regarding the socio-economic impacts of unimproved water sources, offering readers a clearer understanding of the research's importance. Additionally, more detail on the data collection process and criteria for country inclusion would strengthen methodological transparency. A clearer explanation of the Bernoulli model used in spatial scan statistics would further clarify the analytical approach.

While the results are concise, adding visual elements, such as maps and graphs, would improve reader comprehension of the spatial distribution of unimproved water sources. Moreover, a section discussing study limitations, including potential data biases and analytical challenges, would provide a balanced perspective.

Lastly, suggesting future research areas—such as evaluating specific interventions' effectiveness or examining the long-term health impacts of unimproved water sources—could add depth to the conclusion and guide subsequent research.

Overall, this well-structured manuscript addresses a critical public health challenge. With refinements in methodological clarity, background context, and visual presentation, it has the potential to make a meaningful contribution to the understanding and management of unimproved drinking water sources in East Africa.

2. SPECIFIC COMMENTS

2.1 Title

- **Clarity**: The title clearly reflects the focus of the study and its geographical context.
- **Keywords**: Consider including terms like "public health," "water quality," or "spatial analysis" to enhance discoverability.

2.2 ABSTRACT

Background: The introduction effectively highlights the significance of the problem, referencing relevant sources and statistics. However, consider including a brief mention of the specific health impacts associated with unimproved water sources to reinforce the importance of the study.

Methods:

- The use of software tools such as Excel, ArcGIS, and STATA for data management is appropriate for this study. However, would it be beneficial to briefly explain the rationale behind choosing these specific tools and their advantages in spatial analysis? For instance, how does each tool contribute to your work? Do any of them have limitations in covering all aspects of your analysis? While I understand your concerns, could you clarify that I am asking these questions to enhance understanding and not to imply that you should not use these tools?
- Could you please clarify the sample size or scope of the demographic data utilized? Providing this information could enhance the methodology section.

Results:

- **The results are presented concisely, with clear statistical outcomes**. However, could including the specific geographic regions impacted by the primary and secondary clusters help ground the findings more effectively?
- Additionally, have you considered emphasizing the implications of these results, such as potential health impacts or recommendations for local authorities?"

Conclusions:

- **The conclusions offer actionable recommendations, which is excellent.**

  Comment: This opening remark positively highlights the value of actionable recommendations, which is crucial in a research context. It shows that the study has practical implications.
- **It would be helpful to prioritize these recommendations based on their feasibility or expected impact.**

  Comment: This is an excellent suggestion. Prioritizing recommendations would enhance clarity and assist stakeholders in implementing the findings. Consider using a ranking system (e.g., high, medium, low) or providing a brief rationale for the prioritization to guide decision-making.
- **However, mentioning the need for further research in specific areas (e.g., potential interventions or longitudinal studies) could strengthen this section.**

  Comment: This point is crucial for emphasizing the ongoing nature of research in this field. Including specific areas for further research helps to acknowledge gaps in knowledge and suggests directions for future studies. You might also suggest potential methodologies or frameworks that could be used in future research to enhance its applicability.

Overall Impression of Conclusions

The conclusions are strong and provide valuable recommendations. By incorporating prioritization, highlighting areas for further research, and considering potential barriers, you can enhance the effectiveness of this section. Emphasizing stakeholder involvement and providing a call to action will also add to the impact of the conclusions.

Overall Structure and Clarity of Abstract:

- ✓ The structure is logical, progressing from the background to methods, results, and conclusions. However, can you ensure consistency in terminology, particularly regarding "unimproved drinking water sources," and avoid redundancy?

- ✓ Have you considered defining key terms or abbreviations (e.g., LLR, p-values) for readers who may not be familiar with statistical analyses?
- ✓ In the main text, would it be beneficial to reference recent studies or reports that address similar issues in East Africa to provide context for your findings?
- ✓ If not already included in the full manuscript, would adding visual aids (maps, charts) to effectively illustrate spatial distributions and clusters enhance the reader's understanding?
- ✓ In the discussion section, could you address any limitations of your study, such as potential biases in the data source or challenges encountered during data collection?

2.3. INTRODUCTION

Comments and suggestions of Introduction

1. **Access to drinking water is a fundamental necessity and a human right essential for the dignity and health of everyone.**

   Comment: This statement sets a powerful tone by emphasizing the human right to safe drinking water. Consider adding a citation to reinforce this assertion, as it lends credibility to your claim.

2. **As a basic human right clean, safe drinking water that is readily available year-round, in sufficient quantities, uncontaminated by chemicals or dangerous microbes and safe should be available.**

   Comment: The definition of clean, safe drinking water is clear, but the sentence structure is awkward. Consider rephrasing for better clarity: "Clean, safe drinking water, defined as readily available year-round, in sufficient quantities, and free from harmful chemicals or microbes, is a fundamental human right."

3. **According to the World Health Organization and United Nations Children's Fund (WHO/UNICEF) Joint Monitoring Programme, improved drinking-water sources are defined as those that are likely to be protected from external contamination, particularly from fecal matter.**

   Comment: This definition is well-cited and provides essential context. However, consider mentioning the specific year of the report or guidelines to ensure the reader knows the currency of the information.

4. **These sources include household connections, public standpipes, boreholes, protected dug wells, protected springs, and rainwater collection.**

Comment: This list is informative but could benefit from concise phrasing. Consider using a semicolon to separate items for better readability, or even bullet points for clarity.

5. **In contrast, unimproved water sources encompass unprotected wells, unprotected springs, surface water (e.g., rivers, dams, or lakes), vendor-provided water, bottled water (unless water for other uses is available from an improved source), and tanker truck-provided water.**

   Comment: This contrasts well with the previous sentence. However, the phrase "unless water for other uses is available from an improved source" is cumbersome. Consider simplifying it to enhance readability.

6. **WHO and UNICEF report in 2017 reveals that approximately 3 in 10 people worldwide, or 2.1 billion, lack access to safe, readily available water at home.**

   Comment: This statistic is impactful and underscores the significance of the issue. Ensure the citation is formatted consistently to maintain academic rigor.

7. **In addition, around 1 in 4 people lacked safely managed drinking water in their homes in 2020.**

   Comment: This sentence effectively builds on the previous statistic, but it would benefit from a citation for the 2020 data to support your claims.

8. **Half of the world's population that drinks water from contaminated sources is found in Africa, despite the Millennium Development Goals' (MDGs) water target aim being largely successful in various parts of the world.**

   Comment: This is a crucial point that emphasizes the disparity in access to clean water. However, consider rephrasing for clarity: "Despite the MDGs' successes in various regions, Africa still accounts for half of the global population using contaminated water sources."

9. **According to the WHO reports, Sub-Saharan Africa is experiencing the slowest rate of progress in the world.**

   Comment: This statement is strong, but the source should be specified to bolster your argument. Consider providing a citation for the "WHO reports" mentioned here.

10. **Only 54 percent of people used improved drinking water, and only 25 percent in fragile contexts.**

    Comment: While informative, specify what "fragile contexts" refers to for clearer understanding. Are these conflict zones, or economically challenged areas?

11. **A study conducted in 2020 using secondary data estimated that 29% of the 357,979 households in Sub-Saharan Africa used unimproved water for drinking purposes.**

    Comment: This is a significant statistic that supports your argument. However, it would be beneficial to provide a citation for the study to ensure readers can reference it.

12. **In addition, a cross-sectional survey estimated that 38.0% of the population of Sub-Saharan Africa (SSA) reported using an unimproved drinking-water source, although at national levels this was seen to vary between as high as 88.5% in rural Somalia populations to 1.0% in urban populations in Namibia.**

    Comment: The detail on variability among populations is excellent. However, ensure that the statistical data is sourced properly. This information should include a citation for transparency.

13. **In East Africa, the bulk of water at very high risk (68.7%) came from unimproved drinking water sources, including springs and unsecured surface water.**

    Comment: This statistic effectively highlights the risk associated with unimproved water sources. Including a citation would add credibility.

14. **In this study area, the use of unimproved drinking water sources varies from one country to another according to different surveys, with 47% of the population in Ethiopia, 37% in Kenya, 39% in Tanzania, and 41% in Uganda relying on unimproved drinking water sources.**

    Comment: This provides valuable country-specific data but could be better structured. Consider rephrasing for smoother flow: "Surveys indicate variability in the use of unimproved water sources, with 47% of the population in Ethiopia, 37% in Kenya, 39% in Tanzania, and 41% in Uganda relying on such sources."

15. **Climate change, increasing water scarcity, population growth, demographic changes, and urbanization already pose challenges for improved water source supply systems in East Africa.**

    Comment: This sentence outlines critical factors affecting water supply. However, consider using "pose significant challenges" to emphasize the urgency of the issue.

16. **Using unimproved drinking water sources can have significant adverse health effects.**

    Comment: This statement sets up the discussion on health risks well. However, it would be more compelling if it started with "The use of unimproved drinking water sources...".

17. **These include waterborne diseases such as cholera, dysentery, typhoid fever, and hepatitis.**

Comment: This list is strong and pertinent. Consider explaining how these diseases disproportionately affect vulnerable populations to heighten the stakes of your argument.

18. **Additionally, they can cause diarrheal diseases, which can be severe and life-threatening in children and the elderly.**

    Comment: This adds depth to the health implications. However, try rephrasing to eliminate redundancy with the previous sentence regarding waterborne diseases.

19. **Unimproved water sources can also lead to parasitic infections such as Giardia and Cryptosporidium, causing gastrointestinal issues.**

    Comment: This point effectively diversifies the health risks discussed. Ensure proper citation for the data to maintain academic integrity.

20. **Finally, using unimproved water sources can result in skin infections and other dermatological conditions.**

    Comment: This is a valuable addition to the discussion of health impacts, broadening the scope of adverse effects. Again, cite relevant studies to support this claim.

21. **There was a study conducted in 2024 that determine the pooled prevalence of unimproved drinking water source and its associated factors in East Africa but it didn’t show the spatial variation of unimproved drinking water sources in the region.**

    Comment: This statement introduces a relevant study but is confusing due to tense and structure. Consider rephrasing for clarity: "A 2024 study assessed the pooled prevalence of unimproved drinking water sources and their associated factors in East Africa but did not address the spatial variation of these sources."

22. **Therefore, conducting spatial analysis of unimproved water sources will help to identify high-risk areas, understand the spatial distribution patterns, and prioritize regions for interventions by establishing a correlation between the locations of unimproved water sources and incidences of waterborne infections.**

    Comment: This statement effectively outlines the study's purpose, but it’s lengthy and could be broken into shorter sentences for better clarity. Consider: "Thus, conducting a spatial analysis of unimproved water sources will identify high-risk areas and help understand spatial distribution patterns. This analysis can prioritize regions for interventions by correlating the locations of unimproved water sources with incidences of waterborne infections."

23. **Therefore, this study aims to determine the spatial distribution of unimproved drinking water sources in East Africa using demographic and health survey (DHS) data from 12 East African countries.**

Comment: This concluding statement effectively summarizes the aim of the study. Ensure that the importance of this aim is highlighted to create a stronger rationale for the research.

Overall Impression

The introduction provides a solid foundation for the manuscript and establishes the importance of the study's focus on unimproved drinking water sources in East Africa. With adjustments to clarity, structure, and citation consistency, the introduction could significantly enhance the overall impact of the manuscript.

2.4. METHOD

2.4.1 Study Area, Design, and Data Source

1. **The study was conducted in East Africa.**

Comment: A clear opening statement. Consider adding a brief description of why East Africa is of interest for this study.

2. **East Africa is a vast and diverse region encompassing the Horn of Africa and the eastern fringes of the Sahara Desert.**

Comment: Good context. You could enhance this by briefly mentioning the geographical and cultural diversity of the region, which may impact water access and usage.

3. **There are a total of 19 East African countries**

Comment: Ensure that the focus remains on the 12 countries included in the study to avoid confusion. Consider stating the reasons for including only those specific countries.

4. **.A population based crossectional design was conducted.**

Comment: Specify the time frame of data collection for clarity. Additionally, define what a population-based cross-sectional design entails for readers who may not be familiar with the terminology.

5. **Data for this study were derived from the most recent standard Demographic and Health Surveys (DHS) of the 12 East African countries.**

Comment: Good justification for data selection. Mention any criteria for choosing the most recent surveys.

6. **The DHS is a nationally representative survey that provides data for monitoring indicators of population dynamics, nutrition, and health with large sample size.**

Comment: Consider citing specific indicators relevant to your study to provide clarity on the data's utility.

7. **The permission to use the data was granted from DHS program (S1File).**

Comment: Clarify what S1File is, as it might not be obvious to all readers.

8. **Study Population and Sampling**

"DHS used a multistage (two stage) cluster sampling technique to recruit the samples using EAs or (Enumeration Areas) or clusters and households as primary and secondary sampling units, respectively."

Comment: This is well-stated. You might briefly explain why a multistage sampling technique was chosen and its advantages for this type of study.

9. **A total of 206,748 households were sampled in 12 East African countries and 8040 clusters was considered for analysis.**

Comment: Excellent detail on sample size. You may want to note any relevant demographic information about the households sampled (e.g., urban vs. rural).

10. **In DHS there is a probability that some of the regions or countries' were oversampled and some were under-sampled specifically in large countries.**

Comment: Acknowledging potential sampling biases is crucial. Consider discussing how these biases might affect the study's findings.

11. **Therefore, sampling weights were calculated for each household record (HR) of DHS datasets.**

Comment: This is important for maintaining representativeness. A brief explanation of how the weights were calculated or applied could enhance understanding.

3.4.2 Study Variable

1. **According to the WHO/UNICEF joint monitoring programme drinking water sources were categorized as 'unimproved' and 'improved'.**

Comment: This classification is clear. Ensure that the criteria for categorization are well understood and perhaps briefly explain the significance of each category in the context of public health.

3.4.3 Data Management and Analysis

1. **Data management and analysis was performed using Microsoft Excel 2010, STATA 17, ArcGIS 10.7, and Kuldorff's SaTScan 10.1 software.**

Comment: This section is informative. Consider providing a brief rationale for using each software tool, especially if they are used for different types of analyses.

2. **Data management and analysis was performed using Microsoft Excel 2010, STATA version 17, and ArcGIS 10.7 was used for recoding and merging the household recode (HR) datasets from each 12 countries.**

Comment: Ensure that the mention of "12 countries" is consistent throughout the methods. Be specific about the steps taken for data management.

3. **Using the STATA 17 software sampling weights were applied to restore representativeness and adjust for the non-proportional allocation of the sample to strata and regions during the survey process.**

Comment: Consider elaborating on how sampling weights impact the results and the importance of this step in your analysis.

4. **A simple descriptive spatial analysis (Graduated color map) was performed using ArcGIS 10.7.**

Comment: Great mention of visualization. You might want to explain what insights this visualization can provide regarding water sources.

3.4.4 Spatial Autocorrelation (Global Moran's I) Analysis

1. **Examines the degree to which a set of spatial features and their associated data values tend to be clustered together in space...**

Comment: This explanation is clear and helpful for understanding the method's purpose. Consider defining terms like "positive spatial autocorrelation" for clarity.

3.4.5 Cluster and Outlier Analysis

1. **Conducted using the Anselin Local Moran's I statistic..."**

Comment: This is well-explained. Adding an example of how this analysis may be used to inform public health initiatives could enhance its relevance.

3.4.6 Hot Spot and Cold Spot Analysis

1. **Is a spatial analysis technique used to identify areas with statistically significant clusters of high values (hotspots) and low values (cold spots).**

Comment: Good definition. Including an example of how these findings might be utilized in public health practice could enhance reader engagement.

3.4.7 Kriging Interpolation Analysis

1. **Kriging is a more advanced geostatistical interpolation technique that incorporates both the distance and the degree of variation..."**

Comment: This section is well-explained. Providing a brief insight into how Kriging can help in estimating values in under-sampled areas might be useful.

3.4.8 Spatial Scan Statistical Analysis

1. **We used Kuldorff's SaTScan software v.10.1 for spatial scan statistical analysis...**

Comment: This is well-structured, but consider clarifying the significance of using a Bernoulli model in this context and how it fits into the overall analysis.

3.4.9 Ethics Approval and Consent to Participate

1. **This study is a secondary analysis of previously collected and published household survey data.**

Comment: Clear and concise. Consider adding a note on the ethical considerations related to the use of secondary data, particularly how participant anonymity is preserved.

Overall Impression of Methods

The Methods section is comprehensive and provides a detailed overview of the study's design, population, and analysis techniques. Here are a few overall suggestions:

1. **Consistency:** Ensure consistency in terminology and the number of countries discussed throughout the methods.
2. **Clarity:** Some sections could benefit from simplification or clearer definitions of terms for readers who may not be familiar with specific statistical or geographic analyses.
3. **Contextualization:** Where possible, contextualize methods in terms of their relevance to the study's objectives, especially regarding public health implications.

By addressing these comments, you can strengthen the clarity and impact of your Methods section.

2.5. RESULTS

2.5.1 Graduated Color Map

1. The authors effectively present the variation in unimproved drinking water sources across East Africa, highlighting significant clusters of high and low values.

Areas for Improvement: To improve clarity, the authors could specify the exact geographic regions within each country where high or low levels are concentrated, potentially providing sub-regional or city-level insights.

2.5.2 Spatial Autocorrelation

1. The Global Moran's I analysis is appropriately used here, and the authors clearly state the statistical significance of clustering with a z-score and p-value.

Areas for Improvement: Additional interpretation of what the clustering implies in terms of geographic or socio-economic factors could strengthen the analysis. Also, a brief explanation of how spatial autocorrelation is relevant to public health or resource allocation in these regions might enhance understanding for readers.

2.5.3 Cluster and Outlier

1. High-high and low-low clustering in specific countries like Madagascar and Mozambique is well-explained. The choice of visualization through red and blue clusters helps readers quickly interpret areas of concern.

Areas for Improvement: The authors could provide a short explanation of what high-high and low-low clusters mean in terms of population health and access to resources. Also, it may be helpful to discuss potential reasons behind the presence of outliers or extreme clusters in these specific areas.

2.5.4 Hot Spot and Cold Spot

1. This analysis helps pinpoint areas needing immediate attention or improvement in water quality, which is crucial for public health intervention planning.

Areas for Improvement: The authors could add context about why certain countries have more hot spots. For instance, factors such as geographical barriers, local government policies, or population density could be relevant. Additionally, using terms like "regions with high unmet needs for improved water sources" might better contextualize the results for readers focused on public health outcomes.

2.5.5. Kriging Interpolation

1. Kriging is a suitable geostatistical technique here, and the authors have clearly delineated high and low areas of unimproved water sources.

Areas for Improvement: It would be beneficial to include a brief explanation of why Kriging was selected over other interpolation methods, emphasizing its advantages in modeling water source distributions. Providing more detail on how these interpolated regions align with known socio-economic or geographic divisions within East Africa could add depth.

2.5.6 Spatial Scan Statistical

1. SaTScan's spatial analysis is well applied, and identifying primary and secondary clusters with LLR and p-values strengthens the credibility of the findings.

Areas for Improvement: Adding insights into how spatial clusters correlate with health outcomes or potential disease risks related to poor water quality would increase the section's relevance. Furthermore, a summary of the practical implications of detecting primary clusters in Madagascar and Mozambique could provide clearer recommendations for policymakers.

- ❖ Overall, this *Results* section is robust and well-organized but could benefit from additional context connecting spatial analysis findings to health, socioeconomic factors, and regional policy implications. This would help readers interpret the results more holistically.

2.6. DISCUSSION

2.6.1 Interpretation of Results

1. The authors contextualize spatial clusters of unimproved drinking water sources by linking them to climate impacts, poverty, and agricultural reliance, especially in Madagascar and Mozambique, adding depth to the discussion.

To enhance understanding, they could explore how these factors vary regionally, explaining why some areas face greater water access issues. Discussing regional policies and local governance, such as infrastructure investment or environmental protections, could further enrich the analysis of water scarcity across East Africa.

2.6.2. Explanation of Spatial Analysis Techniques

1. The section clearly presents the SaTScan analysis results, highlighting primary and secondary clusters and their links to environmental and socio-political factors across East Africa, which adds credibility and value by showing how spatial analysis identifies high-need areas.

However, a brief comparison of methods, such as the different insights from SaTScan versus hot spot analysis, would be beneficial. For readers less familiar with spatial methods, a short

summary of how these techniques enhance the study's conclusions could clarify their practical value.

2.6.3 Discussion of Regional Challenges

1. The authors effectively address challenges like poverty, climate impacts, and inadequate infrastructure that hinder water access, providing a well-rounded perspective on the factors affecting water quality in the region.

However, statements such as "inadequate water infrastructure" and "lack of political will" would benefit from specific examples or citations to strengthen the argument. Additionally, discussing local adaptations or community-based initiatives to improve water access could offer valuable strategies for stakeholders.

2.6.4 Limitations

1. The authors address limitations clearly, noting the cross-sectional nature of DHS data and potential geographic coverage gaps, which are valid concerns in spatial epidemiology studies. This transparency adds rigor to the analysis.

However, the limitations section could be strengthened by discussing how excluding certain remote or conflict-affected areas might impact the study's generalizability. Additionally, clarifying the limitations due to missing outcome variables in some countries and suggesting solutions for future research (e.g., alternative data sources or mixed methods) would add further depth to this section.

2.6.5 Conclusion

1. The conclusion offers actionable recommendations, focusing on targeted interventions in high-risk areas, infrastructure improvement, and collaborative international support, which adds value for stakeholders.

However, emphasizing the role of local governments and communities in addressing water issues could enhance this section. Including successful case studies or ongoing efforts within East Africa that align with these recommendations would provide a practical perspective and highlight the feasibility of the proposed actions.

2.6.6 Policy and Practice Implications

1. The recommendations are practical and targeted, such as prioritizing hotspot regions for infrastructure improvements and building climate resilience. The call for

collaboration with organizations like WHO, USAID, and development banks aligns with the complex, cross-border challenges of water access in East Africa.

However, including examples of successful partnerships in similar contexts could clarify potential outcomes. Additionally, discussing possible barriers like funding constraints or political instability and suggesting strategies to address them would add depth to the discussion.

Overall Impression

- The *Discussion* is comprehensive and well-structured, linking spatial analysis findings to practical challenges and solutions. Including more specific regional examples and comparing methods could strengthen this section further. Expanding on policy implications with real-world examples and addressing possible barriers to implementation would make the conclusions more actionable and relevant to policymakers.
