# Supplementary material for: Spatial analysis of unimproved drinking water source in East Africa: Using Demographic and Health Survey (DHS) data from 2012–2023
Source: PLoS One. 2025 Mar 20;20(3):e0318189. doi: 10.1371/journal.pone.0318189 (PMC11925304; doi:10.1371/journal.pone.0318189)
Supplement: Attachment — Submitted filename: Comment.pdf [file pone.0318189.s005.pdf]

PLOS ONE

Spatial analysis of unimproved drinking water source in East Africa: Based on demographic and health survey data, 2012-2022

--Manuscript Draft--

**Manuscript Number:**	PONE-D-24-33669
**Article Type:**	Research Article
**Full Title:**	Spatial analysis of unimproved drinking water source in East Africa: Based on demographic and health survey data, 2012-2022
**Short Title:**	Spatial analysis of unimproved drinking water source in East Africa
**Corresponding Author:**	Lidetu Demoze, MPH,Bsc<br>University of Gondar<br>Gondar, ETHIOPIA
**Keywords:**	Spatial, unimproved, drinking water source, East Africa, demographic and health survey data
**Abstract:**	Background: According to the World Health Organization/United Nations International Children's Emergency Fund Joint Monitoring Programme, unimproved drinking water sources encompass unprotected wells, unprotected springs, surface water (e.g., rivers, dams, or lakes), vendor-provided water, bottled water (unless water for other uses is available from an improved source), and tanker truck-provided water. In East Africa, a significant proportion of water at very high risk (68.7%) is sourced from unimproved drinking water sources, including springs and unsecured surface water. This use of unimproved drinking water sources can have significant adverse health effects, such as waterborne diseases. Therefore, this study aimed to determine the spatial distribution of unimproved drinking water sources in East Africa.<br>Methods: A secondary data analysis were conducted using the most recent demographic and health survey data from 12 East African countries. Microsoft Excel 2010, ArcGIS software version 10.7, and STATA 17 were used for data management. The Global Moran's I and spatial scan statistical tests, using the Bernoulli model, were performed to explore the presence of clusters in the study area. These analyses were conducted using ArcGIS software version 10.7 and Kuldorff's SaTScan software v.10.1, respectively.<br>Results: Spatial clusters of unimproved drinking water sources were identified within the study area (Global Moran's I: 0.018, z-score: 87.10, p < 0.05). A total of 167 significant spatial windows containing primary and secondary clusters were identified. The first spatial window contained the primary clusters, while the remaining 166 spatial windows contained secondary clusters. The primary clusters covered nearly all parts of Madagascar and certain coastal regions of Mozambique, exhibiting a Log Likelihood Ratio (LLR) of 5221.25 with a p-value < 0.000. The secondary clusters were found across all 12 countries included in the analysis (Burundi, Comoros, Ethiopia, Kenya, Madagascar, Malawi, Mozambique, Rwanda, Tanzania, Uganda, Zambia, and Zimbabwe), with Log Likelihood Ratios ranging from 11.93 to 3135.38 and p-values < 0.05.<br>Conclusions: This study identified significant clusters, hotspots, and outliers (high-high clusters) of unimproved drinking water sources across various East African countries. To address these issues effectively, priority should be given to the identified clusters, hotspots, and high-high clusters. This involves expanding water treatment facilities, improving water distribution, and protecting drinking water sources. Strengthening regulations, conducting research, and fostering public-private partnerships are also crucial for ensuring sustainable access to clean water. We recommend that East African governments, international organizations such as United Nations agencies (WHO and UNICEF), USAID, other non-governmental organizations, and development banks collaborate to enhance water quality. Their efforts should focus on providing technical assistance, financial support, capacity building, project implementation, advocacy, and financing for drinking water infrastructure in the region.
**Order of Authors:**	Lidetu Demoze, MPH,Bsc
	Kassaw Chekole Adane, MPH,Bsc

Powered by Editorial Manager® and ProduXion Manager® from Aries Systems Corporation

	Jember Azanaw, MSc,Bsc
	Eyob Akalewold, MPH,Bsc
	Tenagne Enawugaw, MSc,Bsc
	Mitkie Tigabie, MSc,Bsc
	Amensisa Hailu Tesfaye, MPH,Bsc
	Gelila Yitageasu, MPH,Bsc
**Additional Information:**	
**Question**	**Response**
**Financial Disclosure**<br><br>Enter a financial disclosure statement that describes the sources of funding for the work included in this submission. Review the submission guidelines for detailed requirements. View published research articles from *PLOS ONE* for specific examples.<br><br>This statement is required for submission and **will appear in the published article** if the submission is accepted. Please make sure it is accurate.<br><br>**Funded studies**<br>Enter a statement with the following details:<br>• Initials of the authors who received each award<br>• Grant numbers awarded to each author<br>• The full name of each funder<br>• URL of each funder website<br>• Did the sponsors or funders play any role in the study design, data collection and analysis, decision to publish, or preparation of the manuscript?<br><br>Did you receive funding for this work?	The author(s) received no specific funding for this work.
**Competing Interests**<br><br>Use the instructions below to enter a competing interest statement for this submission. On behalf of all authors, disclose any competing interests that could be perceived to bias this work—acknowledging all financial support and any other relevant financial or non-financial competing interests.	The authors have declared that no competing interests exist

This statement is required for submission and **will appear in the published article** if the submission is accepted. Please make sure it is accurate and that any funding sources listed in your Funding Information later in the submission form are also declared in your Financial Disclosure statement.<br><br>View published research articles from *PLOS ONE* for specific examples.<br><br>**NO authors have competing interests**<br><br>Enter: *The authors have declared that no competing interests exist.*<br><br>**Authors with competing interests**<br><br>Enter competing interest details beginning with this statement:<br><br>*I have read the journal's policy and the authors of this manuscript have the following competing interests: [insert competing interests here]*<br><br>* typeset	
**Ethics Statement**<br><br>Enter an ethics statement for this submission. This statement is required if the study involved:<br><br>• Human participants<br>• Human specimens or tissue<br>• Vertebrate animals or cephalopods<br>• Vertebrate embryos or tissues<br>• Field research<br><br>Write "N/A" if the submission does not require an ethics statement.<br><br>General guidance is provided below. Consult the submission guidelines for detailed instructions. **Make sure that all**	N/A

**information entered here is included in the Methods section of the manuscript.**<br><br>**Format for specific study types**<br><br>**Human Subject Research (involving human participants and/or tissue)**<br>• Give the name of the institutional review board or ethics committee that approved the study<br>• Include the approval number and/or a statement indicating approval of this research<br>• Indicate the form of consent obtained (written/oral) or the reason that consent was not obtained (e.g. the data were analyzed anonymously)<br><br>**Animal Research (involving vertebrate animals, embryos or tissues)**<br>• Provide the name of the Institutional Animal Care and Use Committee (IACUC) or other relevant ethics board that reviewed the study protocol, and indicate whether they approved this research or granted a formal waiver of ethical approval<br>• Include an approval number if one was obtained<br>• If the study involved *non-human primates*, add *additional details* about animal welfare and steps taken to ameliorate suffering<br>• If anesthesia, euthanasia, or any kind of animal sacrifice is part of the study, include briefly which substances and/or methods were applied<br><br>**Field Research**<br><br>Include the following details if this study involves the collection of plant, animal, or other materials from a natural setting:<br>• Field permit number<br>• Name of the institution or relevant body that granted permission	
**Data Availability**<br><br>Authors are required to make all data underlying the findings described fully available, without restriction, and from the time of publication. PLOS allows rare exceptions to address legal and ethical	Yes - all data are fully available without restriction

concerns. See the PLOS Data Policy and FAQ for detailed information.<br><br>A Data Availability Statement describing where the data can be found is required at submission. Your answers to this question constitute the Data Availability Statement and **will be published in the article**, if accepted.<br><br>**Important:** Stating 'data available on request from the author' is not sufficient. If your data are only available upon request, select 'No' for the first question and explain your exceptional situation in the text box.<br><br>Do the authors confirm that all data underlying the findings described in their manuscript are fully available without restriction?	
**Describe where the data may be found in full sentences. If you are copying our sample text, replace any instances of XXX with the appropriate details.**<br><br>• If the data are **held or will be held in a public repository**, include URLs, accession numbers or DOIs. If this information will only be available after acceptance, indicate this by ticking the box below. For example: *All XXX files are available from the XXX database (accession number(s) XXX, XXX.*).<br>• If the data are all contained **within the manuscript and/or Supporting Information files**, enter the following: *All relevant data are within the manuscript and its Supporting Information files.*<br>• If neither of these applies but you are able to provide **details of access elsewhere**, with or without limitations, please do so. For example:<br><br>*Data cannot be shared publicly because of [XXX]. Data are available from the XXX Institutional Data Access / Ethics Committee (contact via XXX) for researchers who meet the criteria for access to confidential data.*	The data that support the findings of this study have been deposited in the DHS Archive available from https://dhsprogram.com/data/available-datasets.cfm.

The data underlying the results presented in the study are available from (include the name of the third party and contact information or URL).<br>• This text is appropriate if the data are owned by a third party and authors do not have permission to share the data.<br><br>* typeset	
Additional data availability information:	

Powered by Editorial Manager® and ProduXion Manager® from Aries Systems Corporation

Manuscript Click here to access/download;Manuscript;Manuscript.docx

Spatial analysis of unimproved drinking water source in East Africa: Based on demographic and health survey data, 2012-2022

Lidetu Demoze [1*], Kassaw Chekole Adane [3], Jember Azanaw [1], Eyob Akalewold [2], Tenagne Enawugaw [4],Mitkie Tigabie [6], Amensisa Hailu Tesfaye [1, 5], and Gelila Yitageasu [1]

[1] Department of Environmental and Occupational Health and Safety, Institute of Public Health, College of Medicine and Health Sciences, University of Gondar, Gondar, Ethiopia

[2] Department of Epidemiology and Biostatistics, Institute of Public Health, College of Medicine and Health Sciences, University of Gondar, Gondar, Ethiopia

[3] Department of Environmental and Occupational Health and Safety, College of Medicine and Health Sciences, Wollo University, Dessie, Ethiopia

[4] Department of Human physiology, School of Medicine, College of Medicine and Health Sciences, University of Gondar, Gondar, Ethiopia

[5] Institute for Sustainable Futures, University of Technology Sydney, NSW, Sydney, Australia

[6] Department of Medical Microbiology, School of Biomedical and laboratory science, College of Medicine and Health Sciences, University of Gondar, Gondar, Ethiopia

Email address of authors:

Lidetu Demoze: (lidetudemoze12@gmail.com),

Kassaw Chekole Adane: (kassiechiko@gmail.com),

Jember Azanaw: (Jemberazanaw21@gmail.com)

Eyob Akalewold: (eyobmgn@gmail.com),

Tenagne Enawugaw: (tenagneenawgaw27@gmail.com)

Mitkie Tigabie: (mitku1621@gmail.com)

Amensisa Hailu Tesfaye: (amensisahailu@gmail.com)

Gelila Yitageasu: (gelilayitageasu1@gmail.com)

*Corresponding author

Abstract

Background: According to the World Health Organization/United Nations International Children's Emergency Fund Joint Monitoring Programme, unimproved drinking water sources encompass unprotected wells, unprotected springs, surface water (e.g., rivers, dams, or lakes), vendor-provided water, bottled water (unless water for other uses is available from an improved source), and tanker truck-provided water. In East Africa, a significant proportion of water at very high risk (68.7%) is sourced from unimproved drinking water sources, including springs and unsecured surface water. This use of unimproved drinking water sources can have significant adverse health effects, such as waterborne diseases. Therefore, this study aimed to determine the spatial distribution of unimproved drinking water sources in East Africa.

Methods: A secondary data analysis were conducted using the most recent demographic and health survey data from 12 East African countries. Microsoft Excel 2010, ArcGIS software version 10.7, and STATA 17 were used for data management. The Global Moran's I and spatial scan statistical tests, using the Bernoulli model, were performed to explore the presence of clusters in the study area. These analyses were conducted using ArcGIS software version 10.7 and Kuldorff's SaTScan software v.10.1, respectively.

Results: Spatial clusters of unimproved drinking water sources were identified within the study area (Global Moran's I: 0.018, z-score: 87.10, $p < 0.05$). A total of 167 significant spatial windows containing primary and secondary clusters were identified. The first spatial window contained the primary clusters, while the remaining 166 spatial windows contained secondary clusters. The primary clusters covered nearly all parts of Madagascar and certain coastal regions of Mozambique, exhibiting a Log Likelihood Ratio (LLR) of 5221.25 with a p-value < 0.000. The secondary clusters were found across all 12 countries included in the analysis (Burundi, Comoros, Ethiopia, Kenya, Madagascar, Malawi, Mozambique, Rwanda, Tanzania, Uganda, Zambia, and Zimbabwe), with Log Likelihood Ratios ranging from 11.93 to 3135.38 and p-values < 0.05.

Conclusions: This study identified significant clusters, hotspots, and outliers (high-high clusters) of unimproved drinking water sources across various East African countries. To address these issues effectively, priority should be given to the identified clusters, hotspots, and high-high clusters. This involves expanding water treatment facilities, improving water distribution, and

protecting drinking water sources. Strengthening regulations, conducting research, and fostering public-private partnerships are also crucial for ensuring sustainable access to clean water. We recommend that East African governments, international organizations such as United Nations agencies (WHO and UNICEF), USAID, other non-governmental organizations, and development banks collaborate to enhance water quality. Their efforts should focus on providing technical assistance, financial support, capacity building, project implementation, advocacy, and financing for drinking water infrastructure in the region.

Keywords: **Spatial, unimproved, drinking water source, East Africa, demographic and health survey data**

Introduction

Access to drinking water is a fundamental necessity and a human right essential for the dignity and health of everyone(1). As a basic human right clean, safe drinking water that is readily available year-round, in sufficient quantities, uncontaminated by chemicals or dangerous microbes, and safe should be available (2). According to the World health organization and United Nations Children's Fund (WHO/UNICEF) Joint Monitoring Programme, improved drinking-water sources are defined as those that are likely to be protected from external contamination, particularly from fecal matter (3). These sources include household connections, public standpipes, boreholes, protected dug wells, protected springs, and rainwater collection(3). In contrast, unimproved water sources encompass unprotected wells, unprotected springs, surface water (e.g., rivers, dams, or lakes), vendor-provided water, bottled water (unless water for other uses is available from an improved source), and tanker truck-provided water (3-5).

WHO and UNICEF report in 2017 reveals that approximately 3 in 10 people worldwide, or 2.1 billion, lack access to safe, readily available water at home(6).In addition, around 1 in 4 people lacked safely managed drinking water in their homes in 2020(7). Half of the world's population that drinks water from contaminated sources is found in Africa, despite the Millennium Development Goals' (MDGs) water target aim being largely successful in various parts of the world (8).According to the WHO reports Sub-Saharan Africa is experiencing the slowest rate of progress in the world. Only 54 per cent of people used improved drinking water, and only 25 per cent in fragile contexts(7). A study conducted in 2020 using secondary data estimated that 29% of the 357,979 households in Sub-Saharan Africa used unimproved water for drinking purposes (9).

In addition, crossectional survey estimated that in 38.0% of the population of Sub-Saharan Africa SSA (excluding Eritrea and Botswana) reported using an unimproved drinking-water source, although at national levels this was seen to vary between as high as 88.5% in rural Somalia populations to 1.0% in urban populations in Namibia(10).

In East Africa the bulk of water at very high risk (68.7%) came from unimproved drinking water sources, including springs and unsecured surface water(11). In this study area, the use of unimproved drinking water sources varies from one country to another according to different surveys, with 47% of the population in Ethiopia, 37% in Kenya, 39% in Tanzania, and 41% in Uganda relying on unimproved drinking water sources(12). Climate change, increasing water scarcity, population growth, demographic changes and urbanization already pose challenges for improved water source supply systems in East Africa(13). Using unimproved drinking water sources can have significant adverse health effects. These include waterborne diseases such as cholera, dysentery, typhoid fever, and hepatitis. Additionally, they can cause diarrheal diseases, which can be severe and life-threatening in children and the elderly(14, 15). Unimproved water sources can also lead to parasitic infections such as Giardia and Cryptosporidium, causing gastrointestinal issues(16). Finally, using unimproved water source can result in skin infections and other dermatological conditions(14, 15).There was a study conducted in 2024 that determine the pooled prevalence of unimproved drinking water source and its associated factors in East Africa but it didn’t show the spatial variation of unimproved drinking water source in the region(17). Therefore, conducting spatial analysis of unimproved water sources will help to identify high-risk areas, understand the spatial distribution patterns, and prioritize regions for interventions by establishing a correlation between the locations of unimproved water sources and incidences of waterborne infections. Therefore, this study aims to determine the spatial distribution of unimproved drinking water sources in East Africa using demographic and health survey (DHS) data from 12 East African countries.

Method

Study area, design and data source

The study was conducted in East Africa. East Africa is a vast and diverse region encompassing the Horn of Africa and the eastern fringes of the Sahara Desert(18). There are a total of 19 East African

countries that includes Burundi, Comoros, Djibouti, Eritrea, Ethiopia, Kenya, Madagascar, Malawi, Mauritius, Mozambique, Rwanda, Seychelles, Somalia, South Sudan, Sudan, Tanzania, Uganda, Zambia, and Zimbabwe (**Fig.1**). A population based crossectional design was conducted. Data for this study were derived from the most recent standard Demographic and Health Surveys (DHS) of the 12 East African countries: Burundi, Comoros, Ethiopia, Kenya, Madagascar, Mozambique, Malawi, Rwanda, Tanzania, Uganda, Zambia, and Zimbabwe. The data spanned from the 2012 Comoros DHS to the 2023 Tanzania DHS. We excluded some countries because we could not find the outcome variable for them, and some countries did not have DHS. The DHS is a nationally representative survey that provides data for monitoring indicators of population dynamics, nutrition, and health with large sample size. The permission to use the data was granted from DHS program (**S1File**). DHS data is open source and can be retrieved on the DHS website (https://dhsprogram.com/Data/terms-of-use.cfm).

Figure 1: Map of the study area.

Study population and sampling

DHS used a multistage (two stage) cluster sampling technique to recruit the samples using EAs or (Enumeration Areas) or clusters and households as primary and secondary sampling units, respectively. A total of 206,748 households were sampled in 12 East African countries and 8040 clusters was considered for analysis. In DHS there is a probability that some of the regions or countries' were oversampled and some were under-sampled specifically in large countries. Therefore, sampling weights were calculated for each household record (HR) of DHS datasets.

Study variable

Outcome variable

According to the WHO/UNICEF joint monitoring programme drinking water sources were categorized as "unimproved" and "improved". Unimproved water sources include unprotected wells, unprotected springs, surface water (e.g. river, dam or lake), vendor-provided water, bottled water (unless water for other uses is available from an improved source) and tanker truck-provided water. Improved water sources include household connections, public standpipes, boreholes, protected dug wells, protected springs and rainwater collection(3).

Data management and analysis

Data management and analysis was performed using Microsoft Excel 2010, STATA 17, ArcGIS 10.7, and Kuldorff's SaTScan 10.1 software. Microsoft Excel 2010, STATA version 17, and ArcGIS 10.7 was used for recoding and merging the household recode (HR) datasets from each 12 countries. Using the STATA 17 software sampling weights were applied to restore representativeness and adjust for the non-proportional allocation of the sample to strata and regions during the survey process. A simple descriptive spatial analysis (Graduated color map) was performed using ArcGIS 10.7 to identify the geographical dependence and distribution of unimproved water sources in East Africa.

Spatial autocorrelation (Global Moran's I) analysis

Examines the degree to which a set of spatial features and their associated data values tend to be clustered together in space (positive spatial autocorrelation) or dispersed (negative spatial autocorrelation). This analysis helps identify patterns, such as whether high values are found near other high values or if they are spread out across the study area(19).This analysis was conducted using ArcGIS 10.7 software.

Cluster and outlier analysis

Conducted using the Anselin Local Moran's I statistic, is a spatial analysis method used to identify the presence and location of clusters and outliers in spatial data. This analysis helps understand patterns by indicating areas where high or low values cluster together or where outliers significantly differ from their neighbors(20). This analysis was employed using ArcGIS 10.7 software.

Hot spot and cold spot analysis

Is a spatial analysis technique used to identify areas with statistically significant clusters of high values (hotspots) and low values (cold spots). One common method for this type of analysis is the Getis-Ord Gi* statistic in ArcGIS 10.7 (21). This analysis was computed using ArcGIS 10.7 software.

Kriging interpolation analysis

Kriging is a more advanced geostatistical interpolation technique that incorporates both the distance and the degree of variation between known data points to estimate values at unknown points(22, 23). This analysis was conducted using ArcGIS 10.7 software.

Spatial scan statistical analysis.

We used Kuldorff's SaTScan software v.10.1 for spatial scan statistical analysis with the Bernoulli model in order to determine the purely spatial cluster of unimproved drinking water source in East Africa(24). This model is particularly involves case file, control file and geographic coordinate file. Case file a file includes cluster number and the number of unimproved drinking water source. Control file includes same cluster number with case file and the number of improved drinking water source. Geographic coordinate file includes same cluster number with case file and the geographic coordinates(x, y coordinates). A spatial cluster size threshold of 50% of the total population was applied as the upper limit. Primary and secondary clusters were identified and ranked based on their log-likelihood ratio, with corresponding p-values assigned. Therefore, it will show parts of the East Africa region with high unimproved source of drinking water using high log-likelihood ratio (LLR) and p-value less than 0.05 to associate with clusters outside of the window.

Ethics approval and consent to participate

This study is a secondary analysis of previously collected and published household survey data. As such, it did not require independent ethical approval. However, each included survey obtained ethical clearance from respective national review boards, and informed consent was acquired from all participants. The specific consent procedures varied across surveys. Therefore, ethical approval and informed consent are not applicable to this study.

Results

Graduated colour map

The spatial distribution of unimproved drinking water sources across East Africa varied by country. Within countries, the percentage of unimproved water sources per cluster also differed significantly. As illustrated in (**Fig.2**), some areas exhibited high percentage of unimproved water sources (77-100%), while others had very low (0-8%).

Figure 2: Spatial distribution of unimproved drinking water sources across East Africa.

Spatial autocorrelation

Spatial analysis using Global Moran's I revealed a significant clustering of unimproved drinking water sources in East Africa (z-score = 87.10087, $p < 0.0001$). This indicates an extremely low probability (less than 1%) that the observed clustering pattern occurred by chance. The resulting map, with its distinct red and blue clusters, visually confirms this significant spatial pattern (**Fig.3**).

Figure 3: Spatial autocorrelation analysis of unimproved drinking water sources across East Africa.

Cluster and outlier

The map below illustrates areas of extreme concentration (high-high and low-low) of unimproved drinking water sources in East Africa. Red colored regions, classified as high-high clusters, indicate a high prevalence of unimproved water sources, particularly in Madagascar and parts of Mozambique. Conversely, blue-colored areas represent low-low clusters, where unimproved drinking water sources are less common. These low-low clusters encompass portions of Ethiopia, Kenya, Uganda, Tanzania, Rwanda, Burundi, Malawi, Zimbabwe, Mozambique, and Zambia (**Fig.4**).

Figure 4: Cluster and outlier analysis of unimproved drinking water sources across East Africa.

Hot spot and cold spot

Hot spot and cold spot analysis identified Madagascar, Kenya, and Mozambique as regions with high proportions of unimproved drinking water sources. Conversely, Burundi, Comoros, Ethiopia, Malawi, Rwanda, Tanzania, Uganda, Zambia, and Zimbabwe exhibited lower proportions of unimproved water sources within the region (**Fig.5**).

Figure 5: Hotspot and cold spot analysis of unimproved drinking water sources across East Africa.

Kriging interpolation

Spatial Kriging interpolation analysis revealed that areas with higher proportions of unimproved drinking water sources are represented by red on the map, encompassing some parts of Madagascar and Mozambique. Conversely, green colored regions, including Burundi, Comoros, Ethiopia,

Kenya, Malawi, Rwanda, Tanzania, Uganda, Zambia, and Zimbabwe, indicate lower levels of unimproved drinking water sources (**Fig.6**).

Figure 6: Kriging analysis of unimproved drinking water sources across East Africa.

Spatial scan statistical

SaTScan spatial analysis of unimproved drinking water sources in East Africa identified one spatial window containing a primary cluster and 166 spatial windows containing secondary clusters. The primary clusters covers nearly all parts of Madagascar and certain regions of Mozambique. These clusters exhibited a Log Likelihood Ratio (LLR) of 5221.25 with a p-value less than 0.001. The secondary clusters were found in all 12 countries (Burundi, Comoros, Ethiopia, Kenya, Madagascar, Malawi, Mozambique, Rwanda, Tanzania, Uganda, Zambia, and Zimbabwe) included in the analysis with LLR spanned from 11.93 to 3135.38 (**Fig.7**) (**S2File**).

Figure 7: Spatial scan statistical analysis of unimproved drinking water sources across East Africa.

Discussion

This study aimed to investigate the geographical variation of unimproved drinking water sources in East African countries using DHS data. The study indicates a significant spatial variation in the distribution of unimproved drinking water sources across East African countries, as evidenced by cluster and outlier analysis, hot and cold spot identification, Kriging, and SaTScan analysis. SaTScan analysis identified a primary cluster encompassing nearly all of Madagascar and parts of Mozambique, with a Log Likelihood Ratio (LLR) of 5221.25.

This extensive spatial clustering may be attributed to Madagascar's severe water crisis, exacerbated by its extreme vulnerability to climate change(25). The country frequently suffers from the impacts of climate variability, including recent cyclones, which contribute to water scarcity(26). Moreover, Madagascar is highly vulnerable to projected intensifications in flood risks, rainfall, and drought associated with climate change(27). A significant portion of Madagascar's population lives in poverty(28), limiting their ability to invest in water infrastructure or alternative water sources. This is compounded by the fact that Madagascar's economy heavily relies on agriculture, a sector highly vulnerable to water scarcity and climate change(27).The interplay of temporal and spatial rainfall disparities further exacerbates water shortages in the region(29).

Coastal areas of Mozambique were also included within the primary cluster. This might be attributed to upstream over-abstraction, pollution, and the intensified impacts of climate change, such as more severe coastal cyclones, increased flooding, and a higher frequency of drought(30). Similar to Madagascar, a substantial portion of Mozambique population lives below the poverty line, limiting their ability to invest in water infrastructure or access improved water services(31).

The SaTScan analysis identified an additional 166 spatial windows containing secondary clusters distributed across all 12 East African countries: Burundi, Comoros, Ethiopia, Kenya, Madagascar, Malawi, Mozambique, Rwanda, Tanzania, Uganda, Zambia, and Zimbabwe. These clusters exhibited Log Likelihood Ratios (LLRs) ranging from 11.93 to 3135.38. This could be attributed to several factors. The region's arid and semi-arid climate(32) results in water scarcity and irregular rainfall patterns(33). In addition, uneven distribution of water resources, coupled with frequent droughts, floods, and cyclones that disrupt infrastructure and contaminate water sources(34). High poverty rates hinder investments in essential water infrastructure and sanitation, while a significant portion of the population, especially in rural areas, lacks access to basic services including water. Conflict and instability further exacerbate the situation by disrupting water supply systems and impeding development efforts(35). Inadequate water infrastructure, poor sanitation practices, and inefficient water management compounded by corruption and a lack of political will contribute to the problem(36, 37). Furthermore, over-extraction of water, deforestation, and soil erosion contribute to water scarcity and pollution, further compromising the quality of drinking water sources in the region(38).

Limitation of the study

Demographic health survey are typically cross-sectional, providing a snapshot of drinking water status at a specific point in time. Some countries lacked DHS, while others lacked outcome variable this will limit the generalizability across the total 19 East African countries. In addition, there might be geographic gaps in DHS coverage, particularly in remote or conflict-affected areas, limiting the ability to assess drinking water status in these regions.

Conclusion

Despite some progress in achieving the Millennium Development Goals (MDGs) and Sustainable Development Goals (SDGs) related to water targets in several East African countries, the issue of inadequate access to improved drinking water sources remains a significant challenge, as

highlighted by this study. This research identified notable clusters, hotspots, and outliers (high-high clusters) of unimproved drinking water sources across various East African countries. Therefore, priority should be given to the clusters, hotspots, and outliers (high-high clusters) identified in this study to expand water treatment facilities, improve water distribution, and protect water sources. Additionally, building climate resilience, including water harvesting, is essential for ensuring water security. Strengthening regulations, conducting research, and fostering public-private partnerships are crucial for sustainable access to clean water. We recommend that East African governments, international organizations such as United Nations agencies (WHO), USAID, other non-governmental organizations, and development banks collaborate to enhance water quality. Their efforts should focus on providing technical assistance, financial support, capacity building, project implementation, advocacy, and financing for drinking water infrastructure in the region.

Abbreviations

DHS: Demographic and health survey

EA: Enumeration areas

GADM: Global alliance for digital MSMSEs

HR: Household recode

LRR: Log-likelihood ratio

MDGs: Millennium Development Goals

SDGs: Sustainable Development Goals

WHO: World Health Organization

Consent to publication

Not applicable.

Author's contributions

L.D designed the study and develops the proposal. K.C.A and EA worked in data extraction. M.T, J.A and T.E performed analysis and interpretation of the results. L.D, A.H.T and G.Y prepared the manuscript. All the authors read and approved the final manuscript.

Funding

There was no funding source for this study.

Availability of data and materials

Sequence data that support the findings of this study have been deposited in the DHS Archive available from https://dhsprogram.com/data/available-datasets.cfm.

Competing interests

The authors declares that there is no competing interest

Acknowledgments

Authors would like to thank the Demographic and Health Survey (DHS) Program for permitting us to use the DHS dataset.

Supplementary information

S1File: The permission letter to use the data from DHS program.

S2File: Kuldorff's SaTScan analysis result with primary and secondary clusters.

Reference

1. https://www.who.int/data/gho/indicator-metadata-registry/imr-details/8.
2. Goddard JJ, Ray I. Water affordability and human right to water implications in California. 2021;16(1):e0245237.
3. Organization WH. Improved sanitation facilities and drinking-water sources [cited 2024 27]. Available from: https://www.who.int/data/nutrition/nlis/info/improved-sanitation-facilities-and-drinking-water-sources.
4. Shaheed A, Orgill J, Montgomery MA, Jeuland MA, Brown J. Why "improved" water sources are not always safe. Bulletin of the World Health Organization. 2014;92(4):283-9.
5. Gross E EOCaqdfiaupwsiraEfBJoRSJ. Choice and quantity demand for improved and unimproved public water sources in rural areas: Evidence from Benin. Journal of Rural Studies. 2019 Jul 1;69:186-94.
6. Organization WH. https://www.who.int/news/item/12-07-2017-2-1-billion-people-lack-safe-drinking-water-at-home-more-than-twice-as-many-lack-safe-sanitation [updated 12 July 2017 cited 2024 27]. Available from: https://www.who.int/news/item/12-07-2017-2-1-billion-people-lack-safe-drinking-water-at-home-more-than-twice-as-many-lack-safe-sanitation.

7. WHO U. 2021. Available from: https://www.who.int/news/item/01-07-2021-billions-of-people-will-lack-access-to-safe-water-sanitation-and-hygiene-in-2030-unless-progress-quadruples-warn-who-unicef.
8. Matchawe C, Bonny P, Yandang G, Mafo HCY, Nsawir BJ. Water shortages: Cause of water safety in sub-Saharan Africa. Drought-impacts and management: IntechOpen; 2022.
9. Geremew A, Damtew YT. Household water treatment using adequate methods in sub-Saharan countries: evidence from 2013–2016 demographic and health surveys. Journal of Water, Sanitation and Hygiene for Development. 2020;10(1):66-75.
10. Pullan RL, Freeman MC, Gething PW, Brooker SJ. Geographical inequalities in use of improved drinking water supply and sanitation across sub-Saharan Africa: mapping and spatial analysis of cross-sectional survey data. PLoS medicine. 2014;11(4):e1001626.
11. Bogale GG. Hotspots of unimproved sources of drinking water in Ethiopia: mapping and spatial analysis of Ethiopia demographic and health survey Data 2016. BMC Public Health. 2020;20:1-8.
12. WHO U. 2023.
13. Organization WH. Available from: https://www.afro.who.int/health-topics/water.
14. Pal M, Ayele Y, Hadush M, Panigrahi S, Jadhav V. Public health hazards due to unsafe drinking water. Air Water Borne Dis. 2018;7(1000138):2.
15. Clasen T, Pruss-Ustun A, Mathers CD, Cumming O, Cairncross S, Colford Jr JM. Estimating the impact of unsafe water, sanitation and hygiene on the global burden of disease: evolving and alternative methods. Tropical Medicine & International Health. 2014;19(8):884-93.
16. Hikal WM. Parasitic contamination of drinking water and Egyptian standards for parasites in drinking water. Open Journal of Ecology. 2020;10(1):1-21.
17. Terefe B, Jembere MM, Assimamaw NT. Access to drinking safe water and its associated factors among households in East Africa: a mixed effect analysis using 12 East African countries recent national health survey. Journal of health, population, and nutrition. 2024;43(1):72.
18. Terefe B, Jembere MM, Assimamaw NT. Access to drinking safe water and its associated factors among households in East Africa: a mixed effect analysis using 12 East African countries recent national health survey. Journal of Health, Population and Nutrition. 2024;43(1):72.
19. Getis A. Spatial autocorrelation. Handbook of applied spatial analysis: Software tools, methods and applications: Springer; 2009. p. 255-78.
20. Duan L, Xu L, Liu Y, Lee J. Cluster-based outlier detection. Annals of Operations Research. 2009;168:151-68.
21. Chen C-Y, Yang Q-H, editors. Hotspot Analysis of the Spatial and Temporal Distribution of Fires. GISTAM; 2018.
22. Van Beers WC, Kleijnen JP, editors. Kriging interpolation in simulation: a survey. Proceedings of the 2004 Winter Simulation Conference, 2004; 2004: IEEE.
23. Kleijnen JP. Kriging: methods and applications. 2017.
24. Kulldorff M. Spatial scan statistics: models, calculations, and applications. Springer; 1999.
25. Sébastien Desbureaux , Damania R. Rain, forests and farmers: Evidence of drought induced deforestation in Madagascar and its consequences for biodiversity conservation. Biological Conservation 2018 May 1;221:357-64.
26. Harifidy RZ, Hiroshi I, Harivelo RZM, Jun M, Kazuyoshi S, Keiichi M. Assessing future intra-basin water availability in madagascar: Accounting for climate change, population growth, and land use change. Water research. 2024;257:121711.
27. Rigden A GC, Chan D, Huybers P. Climate change linked to drought in Southern Madagascar. npj Climate and Atmospheric Science. 2024 Feb 8;7(1):41.
28. Andrianady J RM. Economic Dynamics in Madagascar: Exploring the Nexus between Growth, Crises, and Political Stability. Crises, and Political Stability (August 16, 2023). 2023 Aug 16.

29. Harrington LJ WP, Pinto I, Ramarosandratana AM, Barimalala R, Vautard R, Philip S, Kew S, Singh R, Heinrich D, Arrighi J. Limited role of climate change in extreme low rainfall associated with southern Madagascar food insecurity, 2019–21. Environmental Research: Climate. 2022 Dec 1;1(2):021003.
30. McCordic C RI, Judyn M, Willis D. The distribution of Cyclone Idai's water impacts in Beira, Mozambique. Disaster Prevention and Management: An International Journal. 2024 Jan 3;33(6):1-5.
31. Giannetti BF LE, Almeida CM, Agostinho F, de Oliveira Neto GC, Lombardi GV. Overcoming poverty traps in Mozambique: Quantifying inequalities among economic, social and environmental capitals. Journal of Cleaner Production. 2023 Jan 10;383:135266.
32. Marigi SN, Njogu AK, Githungo WN. Trends of extreme temperature and rainfall indices for arid and semi-arid lands of south eastern Kenya. Journal of Geoscience and Environment Protection. 2016;4(12):158-71.
33. Fenta AA, Yasuda H, Shimizu K, Haregeweyn N, Kawai T, Sultan D, et al. Spatial distribution and temporal trends of rainfall and erosivity in the Eastern Africa region. Hydrological Processes. 2017;31(25):4555-67.
34. Yadava PK KH, Singh A, Kumar V, Verma S. Impact of climate change on water quality and its assessment. InVisualization Techniques for Climate Change with Machine Learning and Artificial Intelligence. 2023 Jan 1;pp. 39-54Elsevier.
35. Bosch C, Hommann K, Rubio GM, Sadoff C, Travers L. Water, sanitation and poverty. Draft chapter Washington DC: World Bank. 2001.
36. Franz E. Toa Kitu Kidogo: Corruption in East Africa. 2012.
37. Okurut K, Kulabako R, Abbott P, Adogo J, Chenoweth J, Pedley S, et al. Access to improved sanitation facilities in low-income informal settlements of East African cities. Journal of water, sanitation and hygiene for development. 2015;5(1):89-99.
38. Wassie SB. Natural resource degradation tendencies in Ethiopia: a review. Environmental systems research. 2020;9(1):1-29.

Figure

Click here to access/download;Figure;Fig.1.tiff

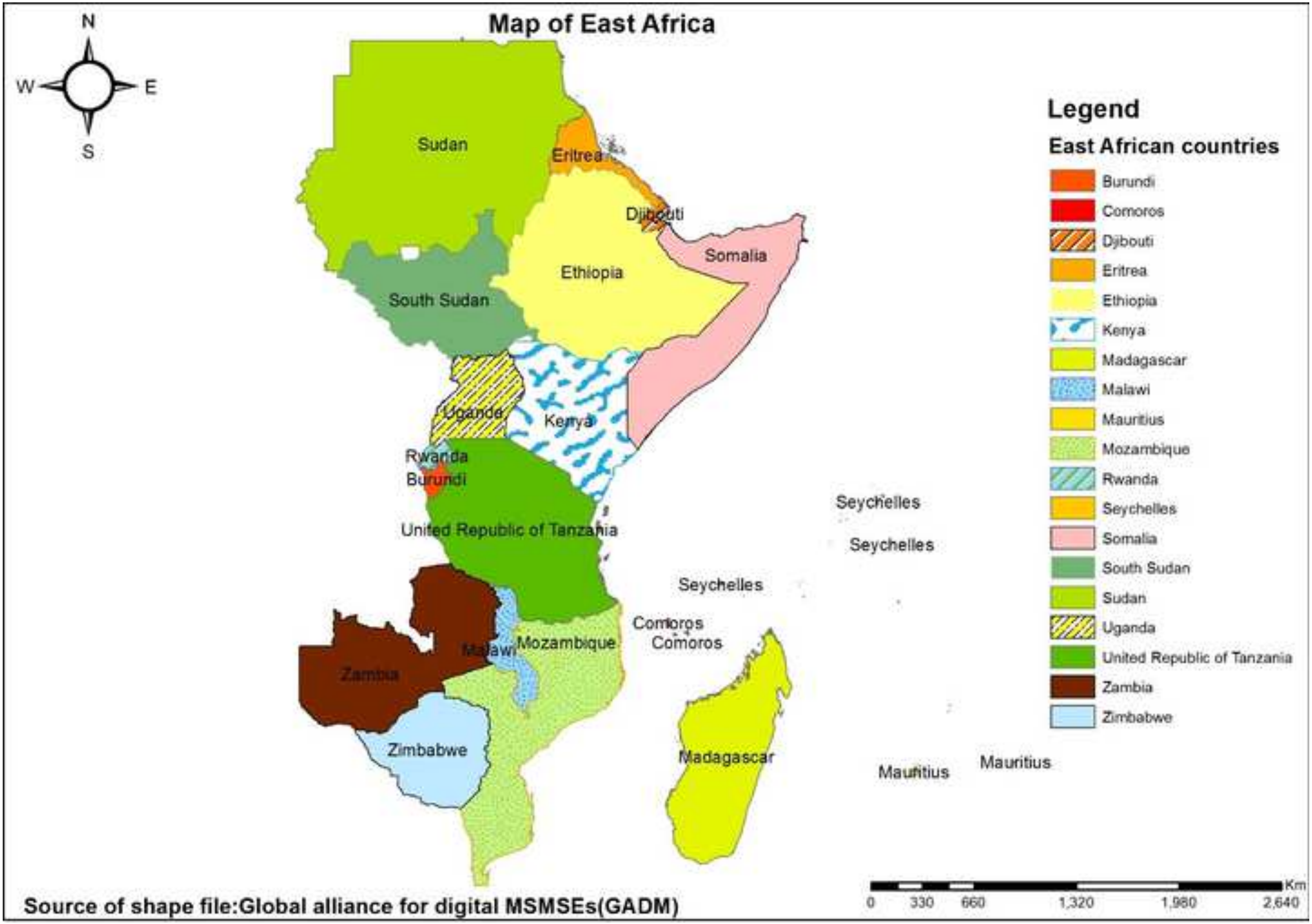

Figure

Click here to access/download;Figure;Fig.2.tiff

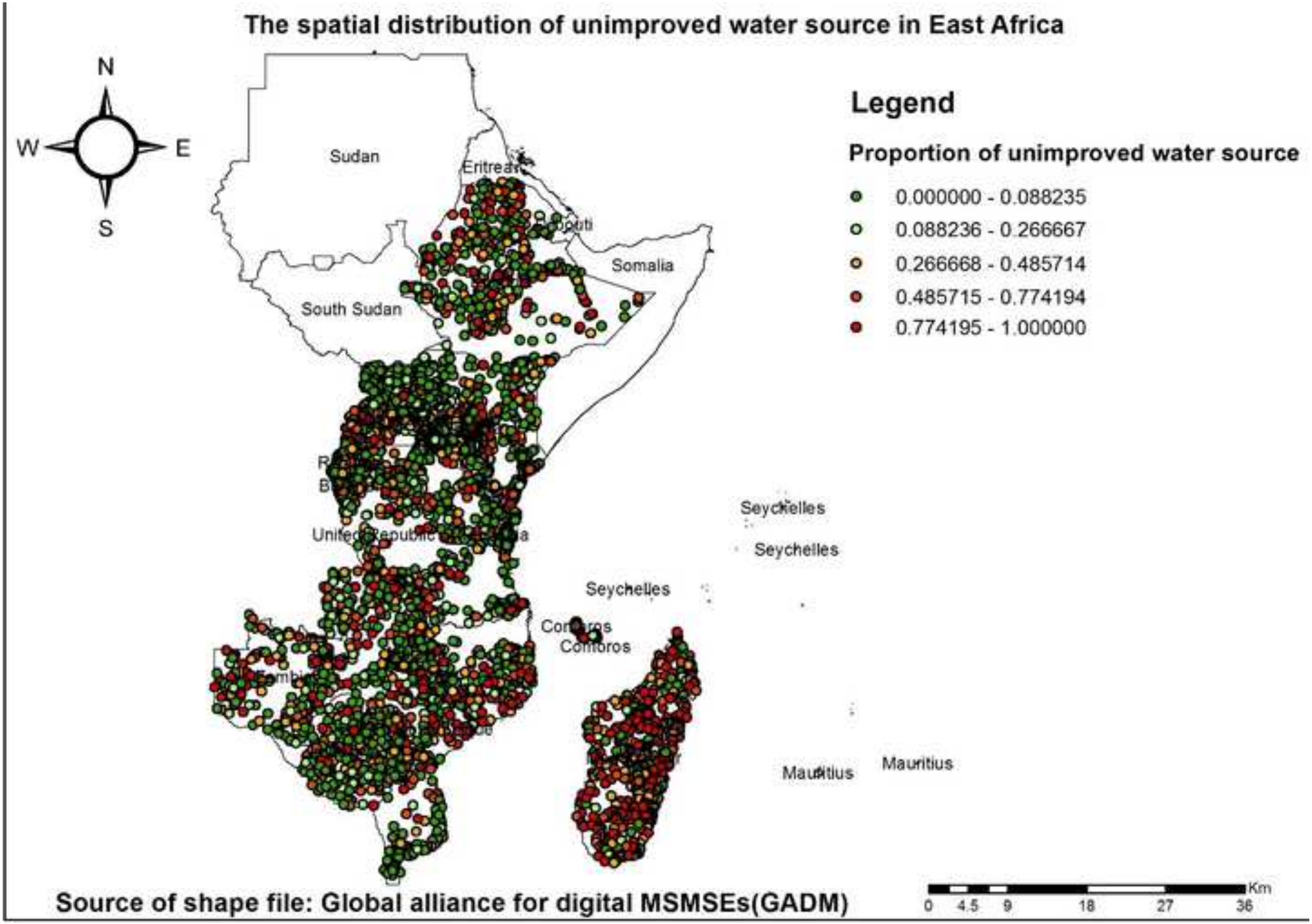

Figure Click here to access/download;Figure;Fig.3.tiff

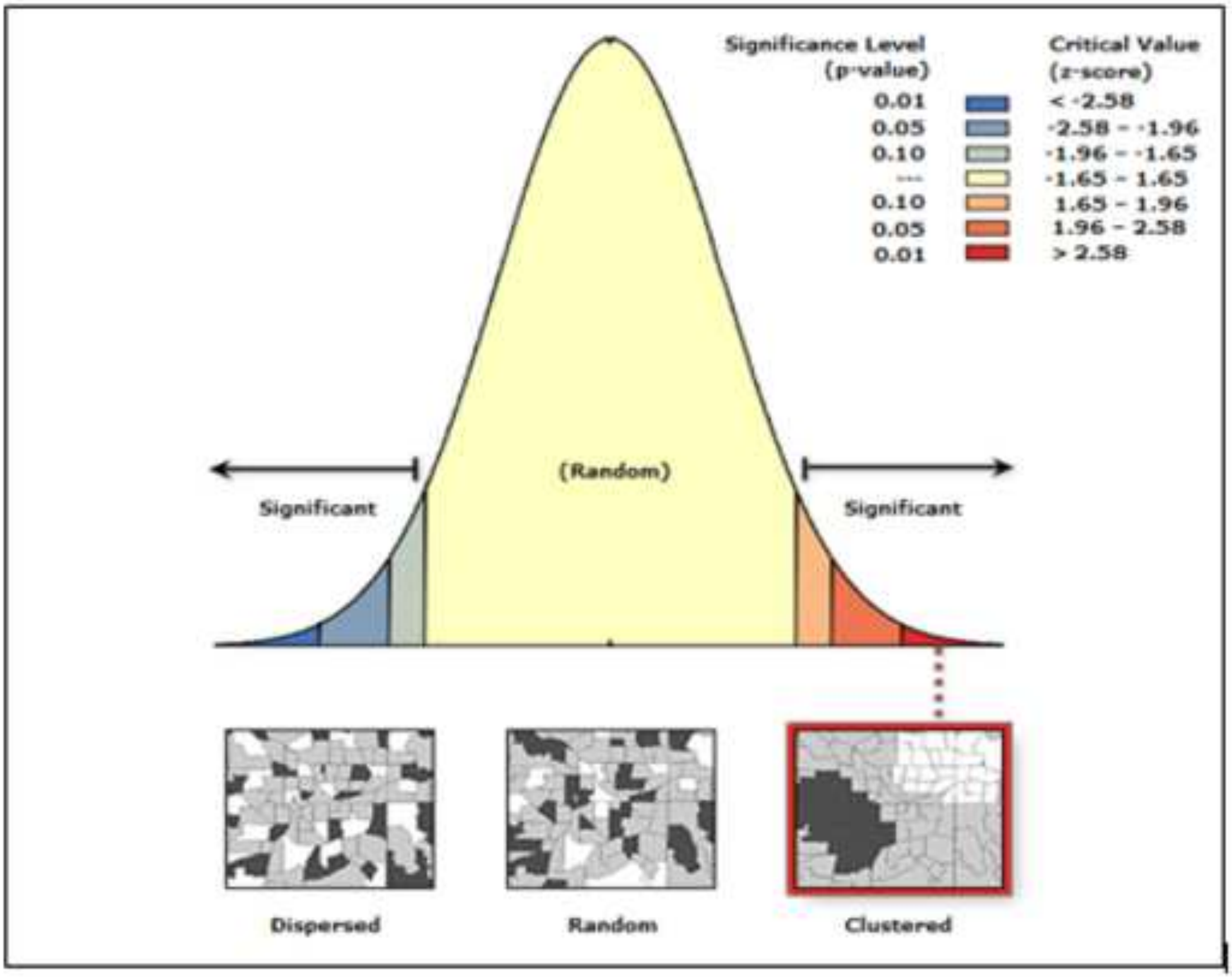

Global Moran's I Summary

Moran's Index:	0.018367
Expected Index:	-0.000125
Variance:	0.000000
z-score:	87.100871
p-value:	0.000000

Figure
Click here to access/download;Figure;Fig.4.tiff

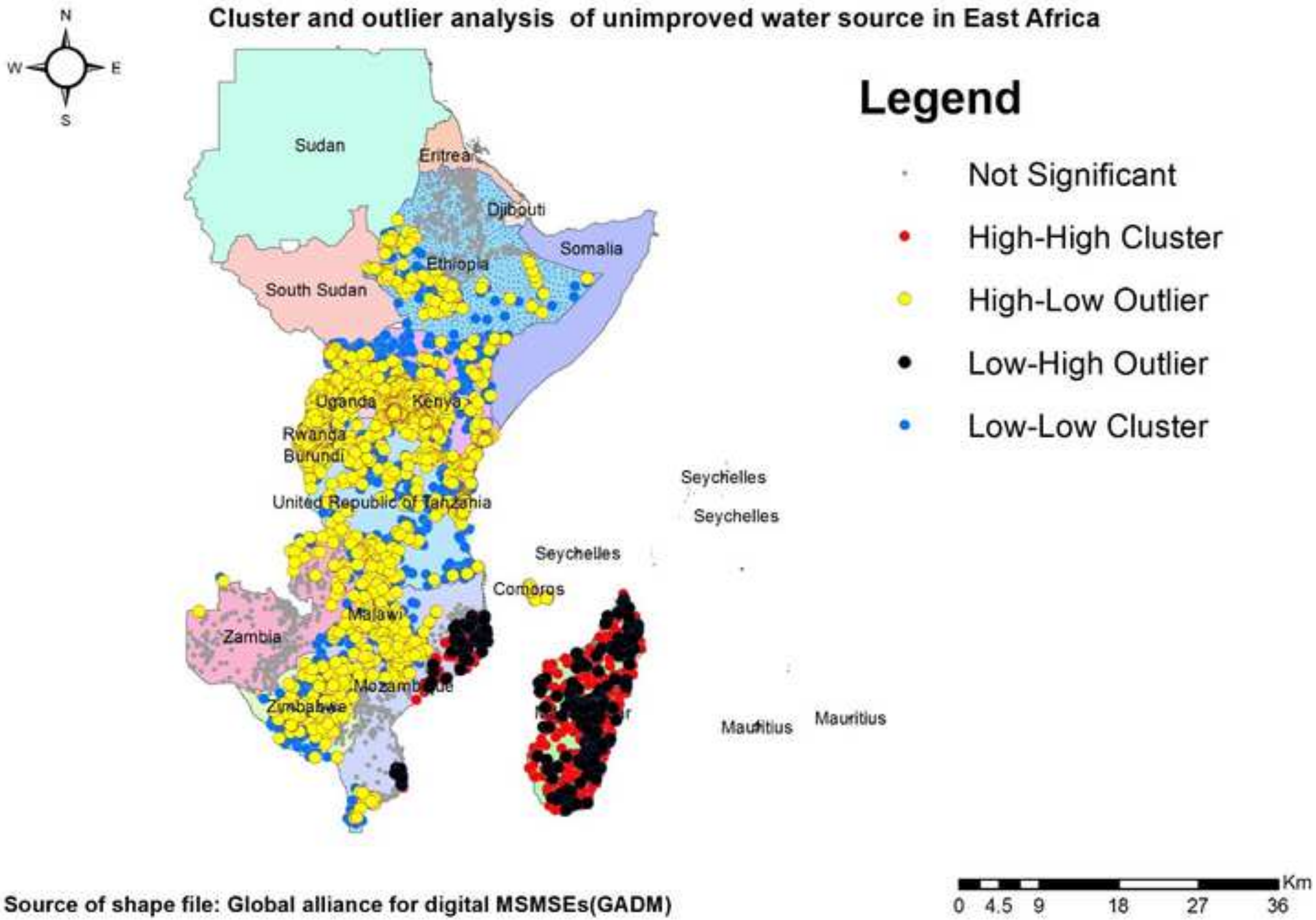

Figure

Click here to access/download;Figure;Fig.5.tiff

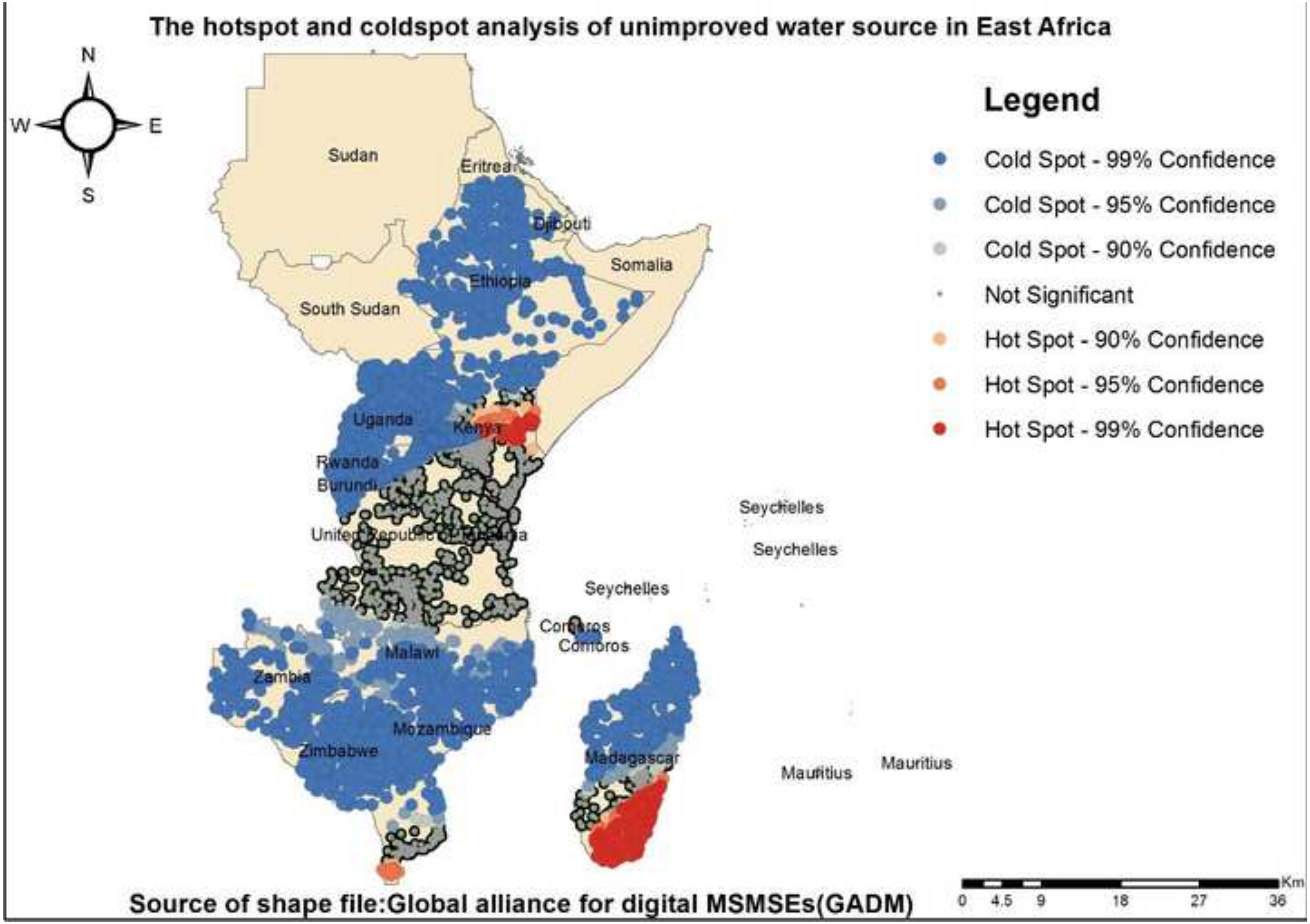

Figure

Click here to access/download;Figure;Fig.6.tiff

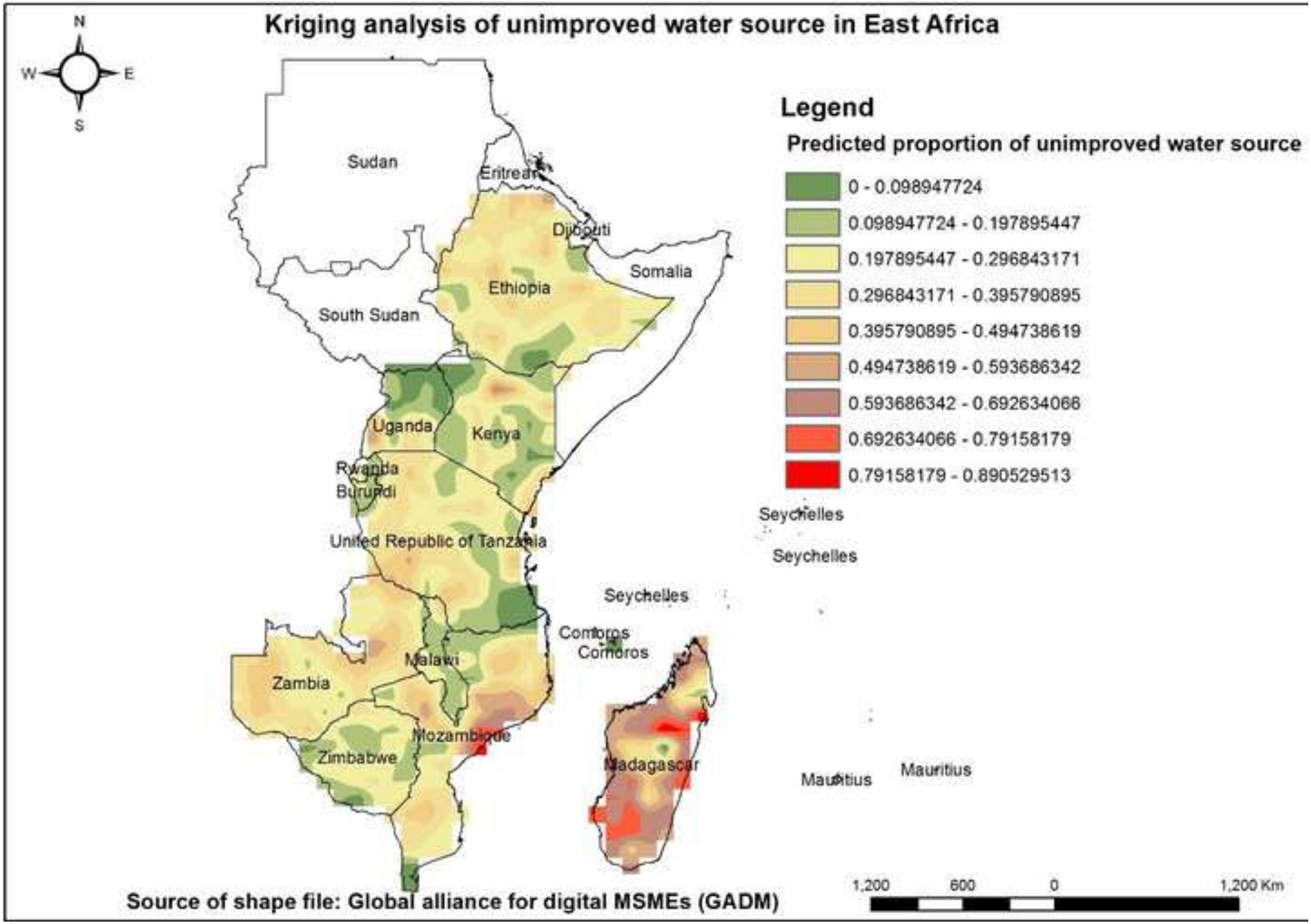

Figure

Click here to access/download;Figure;Fig.7.tiff

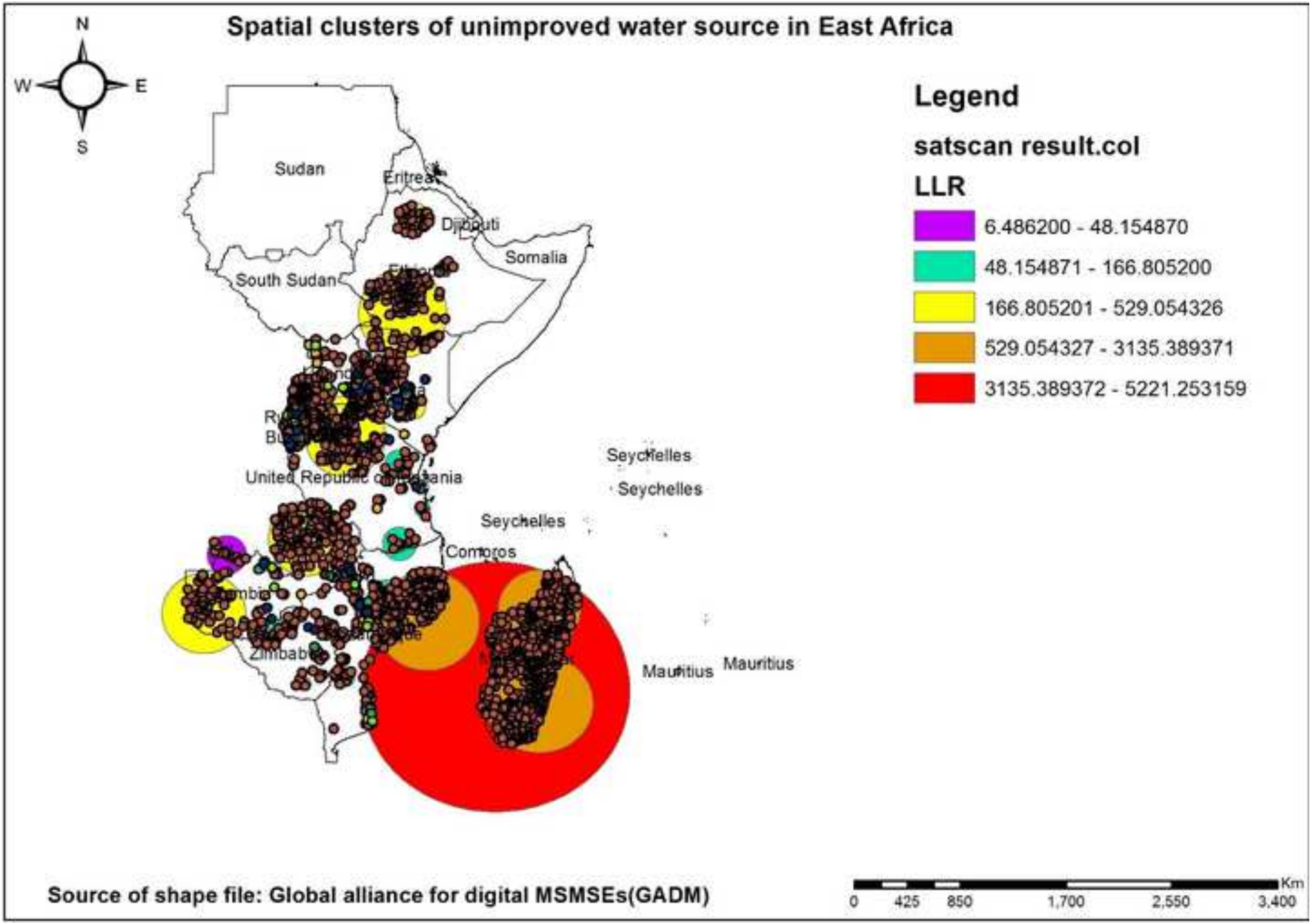

Click here to access/download
Supporting Information
S1File.pdf

Click here to access/download
Supporting Information
S2File.txt
