# Supplementary material for: Spatial analysis of unimproved drinking water source in East Africa: Using Demographic and Health Survey (DHS) data from 2012–2023
Source: PLoS One. 2025 Mar 20;20(3):e0318189. doi: 10.1371/journal.pone.0318189 (PMC11925304; doi:10.1371/journal.pone.0318189)
Supplement: Attachment — Submitted filename: REVIEWER REPORT.docx [file pone.0318189.s001.docx]

this paper add no value to scientific community doing spatial analysis and knowing only the sites of unimproved drinking water source will not provide solutions and another analysis that is multilevel mixed effect model is needed so at this adage I recommend to add multilevel mixed effect model and write and submit again to the journal so that what factors lead to unimproved drinking water source occurrence is very important
