# Supplementary material for: Spatial analysis of unimproved drinking water source in East Africa: Using Demographic and Health Survey (DHS) data from 2012–2023
Source: PLoS One. 2025 Mar 20;20(3):e0318189. doi: 10.1371/journal.pone.0318189 (PMC11925304; doi:10.1371/journal.pone.0318189)
Supplement: Attachment — Submitted filename: Plos One.docx [file pone.0318189.s002.docx]

Dear editor, thank you for the opportunity to invite “Spatial analysis of unimproved drinking water source in East Africa: Based on demographic and health survey data”. The title is very interesting across east Africa. However, the major limitations of these study is without any statistical model to demonstrate any methodological perspectives for unimproved drinking water source. There is no nobility of study. But, only show the spatial patterns in terms of descriptive statistics. So, these study is better to do again in terms of any statistical model considerations. And also, the authors better to consider machine learning approach in addition to any model. Then, this paper requires **major modifications** before publication.

My point of view, the following point is issued.

1. **In abstract part:**

**Background**: background is too large. So, the authors better to minimize the statements again.

**Methods**: well. But, better to add inferential statistics or any statistical model in terms of machine learning approach.

**Results**: good. But, this study is better to consider with statistical model to determine different determinates across east Africa in addition to this work.

**Conclusions**: Conclusions is well organized and a good part of messages related with these results. However, these conclusions part is stronger in terms of statistical model approach in addition to these descriptive nature of the study.

2. **In Introduction part**

I appreciated the structure of each paragraph in terms of global to regional. But, there is some grammatical, punctuation, capitalizations problems.

The gap of study is not enough. There is no novel of study. Then, the authors more clarify consider the gap/ nobility of these study.

3. **Method**

Better to say Material and Methods than “Method”. Study area, design and data source and Study population and sampling is well organized especially in figure.

The authors had outcome variable. But, there was no any statistical model. So, why do not use it???

Generally, the point in methods section was well.

The authors must be check Ethics approval and consent to participate according to journal guidelines. Because these is under declaration sections.

So, the overall expression under the method section is descriptive statistics. **These study is more precise and attractive to consider inferential statistics or any statistical model.**

4. **Results**

The descriptive part in result section is well. But, the authors lost any statistical model under inferential statistics.

5. **Discussion:** The discussion part is more/less better. But, the authors better to compare and contrast these result with previous studies.
6. **Conclusion**: the conclusion part is enough based on authors methodological approaches. But, more strong in terms of statistical model.
7. **Reference:** The reference citation style must be check again according to the journal guidelines.

**General comments**

- These study presents the results of original research across east Africa. But, these study was done in different east Africa countries separately, especially Ethiopia by using different statistical model.
- Results reported have not been published elsewhere.
- The descriptive analyses are performed to a high technical standard and are described in sufficient detail.
- Conclusions are presented in an appropriate fashion and are supported by the data.
- The article is presented in an intelligible fashion and is written in Standard English. But, there is some grammatical, punctuation and capitalization issues in inside these paper.
- The research meets all applicable standards for the ethics of experimentation and research integrity.
- The article adheres to appropriate reporting guidelines and community standards for data availability.
- In my point of view, this paper is near to publication after **major modifications** as some point mentioned in above.
