# Supplementary material for: Spatial analysis of unimproved drinking water source in East Africa: Using Demographic and Health Survey (DHS) data from 2012–2023
Source: PLoS One. 2025 Mar 20;20(3):e0318189. doi: 10.1371/journal.pone.0318189 (PMC11925304; doi:10.1371/journal.pone.0318189)
Supplement: Attachment — Submitted filename: Response to Reviewers.docx [file pone.0318189.s007.docx]

**Responses to the Editors and review’s comments**

**Dear PLOS ONE editorial team,**

Thank you for giving us the opportunity to submit a revised draft of the manuscript, and we would also like to thank you for your crucial comments on our paper (Manuscript ID: **PONE-D-24-33669**). We are very concerned and have combined all the suggested comments provided, which we believe strengthen our paper, and we hope this will render our paper eligible for consideration for publication in your reputed journal. We appreciate the time and effort that you and the reviewers dedicated to providing feedback on our manuscript and are grateful for the insightful comments and valuable improvements to our paper for publication.

The authors would like to inform you that we have addressed the comments and recommendations of the handling editor point by point. In addition, throughout our revision, we made our best corrections too. All changes made to the original version are highlighted using tracking changes and attached as **“Revised Manuscript with Track Changes”**. The unmarked copy of the manuscript is also attached as **“Manuscript”**. In addition, please see below a rebuttal letter that responds to each point raised by the handling editor, and this letter is also attached to the submission as **“Response to Reviewers”**.

**Response to editor’s comments**

**Comments from the handling editor:**

1. [Please ensure that your manuscript meets PLOS ONE's style requirements, including those for file naming. The PLOS ONE style templates can be found at https://journals.plos.org/plosone/s/file?id=wjVg/PLOSOne_formatting_sample_main_body.pdf and](Please%20ensure%20that%20your%20manuscript%20meets%20PLOS%20ONE's%20style%20requirements,%20including%20those%20for%20file%20naming.%20The%20PLOS%20ONE%20style%20templates%20can%20be%20found%20at%20https://journals.plos.org/plosone/s/file?id=wjVg/PLOSOne_formatting_sample_main_body.pdf%20and ) [https://journals.plos.org/plosone/s/file?id=ba62/PLOSOne_formatting_sample_title_authors_affiliations.pdf](https://c05y1x9s.r.us-east-2.awstrack.me/L0/https:%2F%2Fjournals.plos.org%2Fplosone%2Fs%2Ffile%3Fid=ba62%2FPLOSOne_formatting_sample_title_authors_affiliations.pdf/1/010f01924feccf71-39240159-61b1-4787-9585-0c05fd293cdf-000000/WTUrJjPAEwJwGnsQhomYUwX6ns4=179)

**Author’s response:** Dear Editor, thank you very much for your recommendation. We have made the corrections accordingly to meet the journal requirements.

2. We noticed you have some minor occurrence of overlapping text with the following previous publication(s), which needs to be addressed:

Evaluation of World Bank/French Development Agency financed Urban Water Reform Programme in Lagos Water Corporation (2005-2017) - <https://researchportal.bath.ac.uk/en/studentTheses/evaluation-of-world-bankfrench-development-agency-financed-urban->

In your revision ensure you cite all your sources (including your own works), and quote or rephrase any duplicated text outside the methods section. Further consideration is dependent on these concerns being addressed.

**Author’s response:** Dear Editor, thank you very much for your recommendations. We have made the necessary corrections to meet the journal's requirements.

3. We note that Figures 1, 2, 4, 5, 6 and 7 in your submission contain [map/satellite] images which may be copyrighted. All PLOS content is published under the Creative Commons Attribution License (CC BY 4.0), which means that the manuscript, images, and Supporting Information files will be freely available online, and any third party is permitted to access, download, copy, distribute, and use these materials in any way, even commercially, with proper attribution. For these reasons, we cannot publish previously copyrighted maps or satellite images created using proprietary data, such as Google software (Google Maps, Street View, and Earth). For more information, see our copyright guidelines: <http://journals.plos.org/plosone/s/licenses-and-copyright>.

We require you to either (1) present written permission from the copyright holder to publish these figures specifically under the CC BY 4.0 license, or (2) remove the figures from your submission:

a. You may seek permission from the original copyright holder of Figures 1,2,4,5,6 and 7 to publish the content specifically under the CC BY 4.0 license.

We recommend that you contact the original copyright holder with the Content Permission Form (<http://journals.plos.org/plosone/s/file?id=7c09/content-permission-form.pdf>) and the following text:

“I request permission for the open-access journal PLOS ONE to publish XXX under the Creative Commons Attribution License (CCAL) CC BY 4.0 (<http://creativecommons.org/licenses/by/4.0/>). Please be aware that this license allows unrestricted use and distribution, even commercially, by third parties. Please reply and provide explicit written permission to publish XXX under a CC BY license and complete the attached form.”

Please upload the completed Content Permission Form or other proof of granted permissions as an ""Other"" file with your submission.

In the figure caption of the copyrighted figure, please include the following text: “Reprinted from [ref] under a CC BY license, with permission from [name of publisher], original copyright [original copyright year].”

b. If you are unable to obtain permission from the original copyright holder to publish these figures under the CC BY 4.0 license or if the copyright holder’s requirements are incompatible with the CC BY 4.0 license, please either i) remove the figure or ii) supply a replacement figure that complies with the CC BY 4.0 license. Please check copyright information on all replacement figures and update the figure caption with source information. If applicable, please specify in the figure caption text when a figure is similar but not identical to the original image and is therefore for illustrative purposes only.

The following resources for replacing copyrighted map figures may be helpful:

USGS National Map Viewer (public domain): <http://viewer.nationalmap.gov/viewer/>

The Gateway to Astronaut Photography of Earth (public domain): <http://eol.jsc.nasa.gov/sseop/clickmap/>

Maps at the CIA (public domain): <https://www.cia.gov/library/publications/the-world-factbook/index.html> and <https://www.cia.gov/library/publications/cia-maps-publications/index.html>

NASA Earth Observatory (public domain): <http://earthobservatory.nasa.gov/>

Landsat: <http://landsat.visibleearth.nasa.gov/>

USGS EROS (Earth Resources Observatory and Science (EROS) Center) (public domain): [http://eros.usgs.gov/#](http://eros.usgs.gov/)

Natural Earth (public domain): <http://www.naturalearthdata.com/>

**Author’s response:** Dear Editor, thank you very much for your recommendation. We have made the corrections accordingly to meet the journal requirements.

**This is Global Administrative Areas Database (GADM) license**

The Global Administrative Areas Database (GADM) license permits free use of the data for academic and other non-commercial purposes. Redistribution or commercial use, however, requires prior permission. Using GADM data to create maps for publication in academic research articles is permitted, so you may use these maps as figures in articles published by journals like PLoS, Springer Nature, Elsevier, and MDPI. You are allowed (but not required) to publish these articles (and the maps they contain) under an open license, such as CC-BY, as is standard with PLoS journals and may also apply to other open-access publications. Therefore, no permission request is needed.

**Comments from Reviewer #1:**

1. Why you used spatial cluster size threshold of 50%

**Author’s response:** Dear Reviewer, thank you very much for your question. We used spatial cluster size threshold of 50% because using a cluster size threshold of 50% often helps balance sensitivity and specificity. Here’s a breakdown of the rationale:

1. **Reducing False Positives**: A 50% threshold ensures that only areas with relatively high spatial density are marked as clusters, reducing the likelihood of identifying random variations as significant clusters.
2. **Statistical Power**: With a 50% threshold, spatial clusters are defined in a way that maintains sufficient statistical power. It allows clusters that cover a substantial area to be detected without including excessive noise, which could lead to weaker statistical conclusions.
3. **Application-Driven Choice**: The 50% threshold may be chosen based on prior research, empirical evidence, or specific study requirements. In health research or ecological studies, for example, a cluster size of 50% could represent a significant portion of the spatial distribution of events (e.g., disease cases or plant species occurrences), providing relevant insights without diluting the focus.
4. **Interpretability and Visualization**: A threshold like 50% can make clusters easier to interpret and visualize, as it provides a clear cutoff, helping researchers quickly identify areas with a high concentration of features relative to the total study area.

2. In Exclusion criteria You said “We excluded some countries because we could not find the outcome variable for them, and some countries did not have DHS.” What does it mean some countries did not have DHS, does it mean Don’t have DHS data set at all or does not have coordinate spatial file? How you manage zero coordinate shape file?

**Author’s response:** Dear Reviewer, thank you very much for your question. Yes, we excluded some of the countries because they don’t have DHS data set at all like Somalia and South Sudan. In ArcGIS, handling a shapefile with zero-coordinate geometries involves detecting, removing, or repairing these invalid geometries to ensure data integrity. Here are steps you can take to manage zero-coordinate shapefiles in ArcGIS: Select and Delete Zero-Coordinate Geometries: Use the Select by Attributes option to filter features with zero coordinates or empty geometries. In the attribute table, select features where Shape Area = 0 (for polygons) or other identifying criteria for zero-coordinate entries. After selecting, delete the invalid features. Use "Check Geometry": Run this tool to confirm no remaining errors.

3. Which DHS data set type do you use?

**Author’s response:** Dear Reviewer, thank you very much for your question. We used Household recode data set type from DHS.

4. How you measure your outcome variable have you got all categories from WHO/JOINT monitoring program

**Author’s response:** Dear Reviewer, thank you very much for your question. We classified our outcome variable for water sources as "improved" and "unimproved" based on categories from the WHO/UNICEF Joint Monitoring Program. Unimproved water sources include unprotected wells, springs, surface water, vendor-supplied water, and bottled water (if no improved source is available), while improved sources comprise household connections, public standpipes, boreholes, protected wells, springs, and rainwater collection.

5. In the recommendation section, you said, “We recommend that East African governments." What are East African governments? Does it mean the distribution is the same for all east African countries? What was the importance of doing hotspot analysis?

**Author’s response:** Dear Reviewer, thank you very much for your question. Since we identified clusters in all East African countries included in our analysis, we recommend that the governments of Burundi, Comoros, Ethiopia, Kenya, Madagascar, Mozambique, Malawi, Rwanda, Tanzania, Uganda, Zambia, and Zimbabwe focus on improving the quality of drinking water. Although the distribution of unimproved drinking water sources is not the same across all East African countries, hotspot analysis highlights areas with higher-than-expected concentrations of unimproved sources. This allows policymakers, public health officials, and planners to target resources effectively where they are most needed.

**Comments from Reviewer #2:**

1. The phrase “Based on demographic and health survey data” is a bit wordy and could be simplified for conciseness. For example: “Using Demographic and Health Survey (DHS) data from 2012-2022.”

**Author’s response:** Dear Reviewer, thank you very much for your suggestion. We have made the corrections accordingly.

2. Conciseness and Redundancy: Some sentences are too wordy and could be streamlined for clarity. For example: Instead of “A secondary data analysis was conducted using the most recent demographic and health survey data from 12 East African countries,” Consider: “We analyzed recent Demographic and Health Survey (DHS) data from 12 East African countries.” Avoid redundancy. Both “Global Moran’s I” and “spatial scan tests using the Bernoulli model” are mentioned multiple times. Mentioning them once in the methods would suffice.

**Author’s response:** Dear Reviewer, thank you very much for your suggestion. We have made the corrections accordingly.

3. Highlight Key Findings: Rather than listing all results, focus on the most significant insights. Mentioning all 167 spatial windows might overwhelm the reader. A clearer takeaway would be: “Primary clusters were found in Madagascar and coastal Mozambique, with secondary clusters distributed across all 12 countries analyzed.”

**Author’s response:** Dear Reviewer, thank you very much for your suggestion. We have made the corrections accordingly.

4. Avoid overloading with stakeholders: The long list of stakeholders (UN, USAID, development banks, NGOs, etc.) could be condensed to improve readability: “We urge collaboration among governments, international organizations, and NGOs to enhance water infrastructure and ensure sustainable access to clean water.”

**Author’s response:** Dear Reviewer, thank you very much for your suggestion. We have made the corrections accordingly.

5. Introduction: Sentence Structure and Grammar: Avoid run-on sentences to improve clarity. For instance: “According to the WHO and UNICEF Joint Monitoring Programme, improved water sources are defined as those likely protected from external contamination, particularly fecal matter.” can be rephrased for conciseness: “Improved water sources are defined by WHO/UNICEF as those protected from external contamination, especially fecal matter.”

**Author’s response:** Dear Reviewer, thank you very much for your suggestion. We have made the corrections accordingly.

6. Clarify Regional Focus and Data Sources: The transition from global data to East Africa is logical, but a smoother shift will improve flow. Example: “While water access has improved globally, sub-Saharan Africa still faces significant challenges, with East Africa reporting 68.7% of its water from unimproved sources.”

**Author’s response:** Dear Reviewer, thank you very much for your suggestion. We have made the corrections accordingly.

7. Research Objective and Rationale: The objective could be more concisely stated: “This study aims to map the spatial distribution of unimproved drinking water sources across 12 East African countries using DHS data from 2012-2022.”

**Author’s response:** Dear Reviewer, thank you very much for your suggestion. We have made the corrections accordingly.

8. Limitations of Prior Studies: The mention of the 2024 study on pooled prevalence is valuable, but it should clarify more succinctly how this study differs: “Previous studies focused on pooled prevalence and associated factors but lacked insights into spatial variation, which this study aims to address.”

**Author’s response:** Dear Reviewer, thank you very much for your suggestion. We have made the corrections accordingly.

9. Method: Study Design and Region: Including the exclusion criteria for some countries strengthens clarity. However, it may be helpful to explicitly mention which excluded countries lacked DHS data or outcome variables.

**Author’s response:** Dear Reviewer, thank you very much for your suggestion. We have made the corrections accordingly.

10. Study Population and Sampling: The total sample size (206,748 households) is reported clearly, which helps convey the study's scale. However, providing more detail on how the clusters (8,040) are distributed across the 12 countries would enhance transparency.

**Author’s response:** Dear Reviewer, thank you very much for your suggestion. We have made the corrections accordingly.

11. Data Management and Software: The use of various software tools (Excel, STATA, ArcGIS, and SaTScan) is comprehensive. A small improvement would be to describe briefly the purpose of each tool. For example: Microsoft Excel: Used for initial data cleaning. STATA: For statistical weighting and merging data files. ArcGIS: For geospatial analysis and mapping. SaTScan: To identify spatial clusters using statistical models. Including version numbers of the software is good practice. Make sure the spellings are consistent (e.g., “Kuldorff’s SaTScan”).

**Author’s response:** Dear Reviewer, thank you very much for your suggestion. We have made the corrections accordingly.

12. Spatial Analysis Methods: Moran’s I and Anselin Local Moran’s I: These are well explained. Clarifying the interpretation of positive/negative autocorrelation for a non-technical audience might improve accessibility. Hotspot/Coldspot Analysis: The Getis-Ord Gi* method is mentioned appropriately, but it may help to specify why this method was chosen over others (e.g., due to its robustness in spatial clustering). Kriging: The description is concise, but it may be helpful to mention if ordinary or universal Kriging was used, as they differ slightly in assumptions and applications.

**Author’s response:** Dear Reviewer, thank you very much for your suggestion. We have made the corrections accordingly.

13. Results: Graduated Color Map: Potential Improvement: Add quantitative summary statistics (e.g., the median or range of proportions per country) to supplement the map. Also, briefly describe any patterns by geography, like whether coastal areas tend to have better access than inland regions. There is a country or a region that has no data; please put those regions that have no data on the legend for figure two.

**Author’s response:** Dear Reviewer, thank you very much for your suggestion. We have made the corrections accordingly.

14. Kriging Interpolation Analysis: Potential Improvement: Mention limitations of Kriging, such as areas where data might be sparse and the predictions less reliable. Also, consider discussing areas where predictions diverge from expected trends, prompting further investigation.

**Author’s response:** Dear Reviewer, thank you very much for your suggestion. We have made some corrections accordingly. There are no areas where predictions diverge from expected trends.

15. Spatial Scan Statistical Analysis (SaTScan): Potential Improvement: Explain the significance of primary and secondary clusters. For example, what distinguishes the primary cluster in Madagascar from the other regions? Also, briefly discuss why certain secondary clusters may require targeted but less intensive interventions.

**Author’s response:** Dear Reviewer, thank you very much for your suggestion. We have made the corrections accordingly.

16. Linking Results to Causes: Potential Improvement: Strengthen the connection between your statistical results and the identified causes. For instance, mention how the LLRs reflect the severity of water issues or highlight how coastal vs. inland disparities impact the results.

**Author’s response:** Dear Reviewer, thank you very much for your suggestion. We have made the corrections accordingly.

17. Citing data sources and evidence: Potential Improvement: Expressly relate your discussion points to the spatial analysis findings. For example, link specific clusters to rainfall patterns or flood-prone areas, referring to maps or figures.

**Author’s response:** Dear Reviewer, thank you very much for your suggestion. We have made the corrections accordingly.

18. Secondary Clusters: Further Exploration: Potential Improvement: Provide more detailed insights into variations across secondary clusters. Are there clusters that are unexpectedly severe or others that show gradual improvement? A more granular discussion can deepen the reader’s understanding.

**Author’s response:** Dear Reviewer, thank you very much for your suggestion. We have made the corrections accordingly.

19. Balanced Discussion: Potential Improvement: Include positive developments, if applicable. For example, mention countries or regions showing progress or resilience in managing unimproved water sources. This adds nuance and balance to the discussion.

**Author’s response:** Dear Reviewer, thank you very much for your suggestion. We have made the corrections accordingly.

20. Conclusion: The conclusion effectively summarizes the findings and offers useful recommendations. However, it can be improved by: Reducing redundancy, providing more region-specific recommendations, balancing the tone with some acknowledgment of progress, Offering clearer roles for stakeholders, and Emphasizing the importance of ongoing monitoring and adaptation. These improvements will make the conclusion more impactful, actionable, and aligned with the study’s insights.

**Author’s response:** Dear Reviewer, thank you very much for your suggestion. We have made the corrections accordingly.

21. What is the strength of your article? Please mention.

**Author’s response:** Dear Reviewer, thank you very much for your suggestion. We have made the corrections accordingly.

**Comments from Reviewer #3:**

1. This paper add no value to scientific community doing spatial analysis and knowing only the sites of unimproved drinking water source will not provide solutions and another analysis that is multilevel mixed effect model is needed so at this adage I recommend to add multilevel mixed effect model and write and submit again to the journal so that what factors lead to unimproved drinking water source occurrence is very important.

**Author’s response:** Dear Reviewer, thank you very much for your suggestion. However, a study has already been conducted using a multilevel mixed-effects model to determine the factors contributing to the occurrence of unimproved drinking water sources in East Africa, utilizing DHS data. Here is the link to the study: <https://jhpn.biomedcentral.com/articles/10.1186/s41043-024-00562-y>.

The **spatial analysis of unimproved drinking water sources in East Africa** could offer significant contributions to the scientific community, even when a multilevel mixed-effects model study has already been conducted. Spatial analysis focuses on the geographic distribution and patterns of unimproved water sources, revealing regional disparities, clustering, or trends that may not be apparent through purely statistical models. This approach can help identify high-risk areas for targeted interventions. Additionally, spatial analysis often includes the creation of maps and visual representations that make data more accessible and actionable for policymakers and planners, serving as valuable tools for communication and decision-making. Unlike multilevel mixed-effects models, which emphasize hierarchical data structures, spatial analysis integrates geographic variables such as elevation, land use, or proximity to improved sources to better understand environmental and locational factors influencing water source quality. It also explores spatial autocorrelation and dependencies, highlighting spillover effects or shared vulnerabilities that could influence water access. By combining findings from spatial analysis with those from multilevel models, researchers can achieve a more holistic understanding, with the former providing a regional or national layer of insight to complement household or community-level variability explored in the latter. This integrated perspective can inform policy by identifying hotspots and spatial trends, enabling geographically targeted interventions, infrastructure investments, and resource allocation to reduce reliance on unimproved water sources. Despite overlapping areas, spatial analysis emphasizes geography and visualization, providing a unique and actionable dimension to understanding the distribution and risks associated with unimproved drinking water sources.

**Comments from Reviewer #4:**

1. Dear editor, thank you for the opportunity to invite “Spatial analysis of unimproved drinking water source in East Africa: Based on demographic and health survey data”. The title is very interesting across east Africa. However, the major limitations of these study is without any statistical model to demonstrate any methodological perspectives for unimproved drinking water source. There is no nobility of study. But, only show the spatial patterns in terms of descriptive statistics. So, these study is better to do again in terms of any statistical model considerations. And also, the authors better to consider machine learning approach in addition to any model. Then, this paper requires major modifications before publication.

**Author’s response:** Dear Reviewer, thank you very much for your suggestion. However, a study has already been conducted using a multilevel mixed-effects model to determine the factors contributing to the occurrence of unimproved drinking water sources in East Africa, utilizing DHS data. Here is the link to the study: <https://jhpn.biomedcentral.com/articles/10.1186/s41043-024-00562-y>.

In addition, the advantage of spatial analysis over a machine learning approach lies in its ability to explicitly address the geographic context, spatial relationships, and patterns within the data. Spatial analysis is specifically designed to study the geographic distribution of phenomena and incorporates spatial relationships such as proximity, clustering, and dispersion, which machine learning models often overlook unless explicitly adapted for spatial data. It identifies spatial autocorrelation and dependence, providing insights into geographic processes. Additionally, spatial analysis offers greater interpretability through visual outputs like maps, making it easier for policymakers, planners, and stakeholders to understand and apply the findings in decision-making. In contrast, machine learning models, especially complex ones like deep learning, can act as "black boxes," making it challenging to explain the relationships between inputs and outputs. Spatial analysis also directly integrates variables such as distance, elevation, and clustering, whereas machine learning typically requires preprocessing or feature engineering to incorporate spatial data effectively. Furthermore, spatial analysis emphasizes understanding the underlying geographic processes and relationships driving observed patterns, while machine learning often focuses on prediction or classification accuracy, potentially neglecting causal or contextual geographic understanding. The central role of maps and spatial visualization in spatial analysis makes it an effective tool for communicating findings to non-technical audiences, whereas machine learning outputs often require additional steps to translate into actionable insights. Lastly, spatial analysis is specifically designed to address spatial bias or heterogeneity, ensuring that regional differences or local effects are appropriately accounted for, unlike machine learning models, which may inadvertently produce biased results if spatial dependencies are not properly addressed.

2. In abstract part: Background: background is too large. So, the authors better to minimize the statements again.

**Author’s response:** Dear Reviewer, thank you very much for your suggestion. We have made the corrections accordingly.

3. Methods: well. But, better to add inferential statistics or any statistical model in terms of machine learning approach.

**Author’s response:** Dear Reviewer, thank you very much for your suggestion. Spatial analysis lies in its ability to explicitly address the geographic context, spatial relationships, and patterns within the data. Spatial analysis is specifically designed to study the geographic distribution of phenomena and incorporates spatial relationships such as proximity, clustering, and dispersion, which machine learning models often overlook unless explicitly adapted for spatial data. Therefore, there is no need to conduct a spatial analysis. However, if you have any questions about the spatial analysis methodology, feel free to ask.

4. Results: good. But, this study is better to consider with statistical model to determine different determinates across east Africa in addition to this work.

**Author’s response:** Dear Reviewer, thank you very much for your suggestion. Spatial analysis lies in its ability to explicitly address the geographic context, spatial relationships, and patterns within the data. Spatial analysis is specifically designed to study the geographic distribution of phenomena and incorporates spatial relationships such as proximity, clustering, and dispersion, which machine learning models often overlook unless explicitly adapted for spatial data. Therefore, there is no need to conduct a spatial analysis. However, if you have any questions about the spatial analysis methodology, feel free to ask.

5. Conclusions: Conclusions is well organized and a good part of messages related with these results. However, these conclusions part is stronger in terms of statistical model approach in addition to these descriptive nature of the study.

**Author’s response:** Dear Reviewer, thank you very much for your suggestion. Spatial analysis lies in its ability to explicitly address the geographic context, spatial relationships, and patterns within the data. Spatial analysis is specifically designed to study the geographic distribution of phenomena and incorporates spatial relationships such as proximity, clustering, and dispersion, which machine learning models often overlook unless explicitly adapted for spatial data. Therefore, there is no need to conduct a spatial analysis. However, if you have any questions about the spatial analysis methodology, feel free to ask.

6. I appreciated the structure of each paragraph in terms of global to regional. But, there is some grammatical, punctuation, capitalizations problems. The gap of study is not enough. There is no novel of study. Then, the authors more clarify consider the gap/ nobility of these study.

**Author’s response:** Dear Reviewer, thank you very much for your suggestion. We have made some corrections accordingly.

7. Method Better to say Material and Methods than “Method”. Study area, design and data source and Study population and sampling is well organized especially in figure. The authors had outcome variable. But, there was no any statistical model. So, why do not use it???

**Author’s response:** Dear Reviewer, thank you very much for your suggestion. We have made some corrections accordingly. However, a study has already been conducted using a multilevel mixed-effects model to determine the factors contributing to the occurrence of unimproved drinking water sources in East Africa, utilizing DHS data. We have also mentioned this study in the background section. Here is the link to the study: <https://jhpn.biomedcentral.com/articles/10.1186/s41043-024-00562-y>.

In addition there are several studies that used only a spatial analysis to determine the spatial variation. Here the link for these studies: <https://link.springer.com/article/10.1186/S12889-020-08957-2> and <https://www.cell.com/heliyon/fulltext/S2405-8440(20)30673-3>

8. Generally, the point in methods section was well. The authors must be check Ethics approval and consent to participate according to journal guidelines. Because these is under declaration sections. So, the overall expression under the method section is descriptive statistics. These study is more precise and attractive to consider inferential statistics or any statistical model.

**Author’s response:** Dear Reviewer, thank you very much for your suggestion. We have made some corrections accordingly.

9. **Results**. The descriptive part in result section is well. But, the authors lost any statistical model under inferential statistics. **Discussion:** The discussion part is more/less better. But, the authors better to compare and contrast these result with previous studies. **Conclusion**: the conclusion part is enough based on authors methodological approaches. But, more strong in terms of statistical model. **Reference**: The reference citation style must be check again according to the journal guidelines.

**Author’s response:** Dear Reviewer, thank you very much for your suggestion. We have made some corrections accordingly. We address some of the questions mentioned in the responses above.

10. General comments: These study presents the results of original research across east Africa. But, these study was done in different east Africa countries separately, especially Ethiopia by using different statistical model. Results reported have not been published elsewhere. The descriptive analyses are performed to a high technical standard and are described in sufficient detail. Conclusions are presented in an appropriate fashion and are supported by the data. The article is presented in an intelligible fashion and is written in Standard English. But, there is some grammatical, punctuation and capitalization issues in inside these paper. The research meets all applicable standards for the ethics of experimentation and research integrity. The article adheres to appropriate reporting guidelines and community standards for data availability. In my point of view, this paper is near to publication after major modifications as some point mentioned in above.

**Author’s response:** Dear Reviewer, thank you very much for your comments.

**Comments from Reviewer #5:**

1. Review Comments to the Author: The manuscript titled "Spatial Analysis of Unimproved Drinking Water Sources in East Africa: Based on Demographic and Health Survey Data, 2012-2022" addresses a critical public health issue. The analysis of unimproved drinking water sources is timely and relevant, given the significant health implications associated with such sources in East Africa. Below are my detailed comments and suggestions for improvement

**Author’s response:** Dear Reviewer, thank you very much for your comments.

2. Technical Soundness: Overall, the manuscript presents a technically sound piece of research. The authors employ robust statistical methods, including Global Moran's I and spatial scan statistics, which enhance the validity of the findings. However, some aspects of the methodology could be clarified, particularly regarding the Bernoulli model used in the spatial scan statistical tests. A more detailed explanation would improve the reader's understanding.

**Author’s response:** Dear Reviewer, thank you very much for your suggestion. We have made the corrections accordingly.

3. Keywords: Consider including terms like "public health," "water quality," or "spatial analysis" to enhance discoverability.

**Author’s response:** Dear Reviewer, thank you very much for your suggestion. We have made the corrections accordingly.

4. The use of software tools such as Excel, ArcGIS, and STATA for data management is appropriate for this study. However, would it be beneficial to briefly explain the rationale behind choosing these specific tools and their advantages in spatial analysis? For instance, how does each tool contribute to your work? Do any of them have limitations in covering all aspects of your analysis? While I understand your concerns, could you clarify that I am asking these questions to enhance understanding and not to imply that you should not use these tools?

**Author’s response:** Dear Reviewer, thank you very much for your suggestion. We have made the corrections accordingly.

5. Could you please clarify the sample size or scope of the demographic data utilized? Providing this information could enhance the methodology section.

**Author’s response:** Dear Reviewer, thank you very much for your suggestion. We have made the corrections accordingly.

6. However, could including the specific geographic regions impacted by the primary and secondary clusters help ground the findings more effectively? Additionally, have you considered emphasizing the implications of these results, such as potential health impacts or recommendations for local authorities?"

**Author’s response:** Dear Reviewer, thank you very much for your suggestion. We have made some corrections accordingly. Including health implications and recommendations in the results section of the abstract does not seem feasible.

7. It would be helpful to prioritize these recommendations based on their feasibility or expected impact. This is an excellent suggestion. Prioritizing recommendations would enhance clarity and assist stakeholders in implementing the findings. Consider using a ranking system (e.g., high, medium, low) or providing a brief rationale for the prioritization to guide decision-making.

**Author’s response:** Dear Reviewer, thank you very much for your suggestion. We have made the corrections accordingly.

8. However, mentioning the need for further research in specific areas (e.g., potential interventions or longitudinal studies) could strengthen this section. Comment: This point is crucial for emphasizing the ongoing nature of research in this field. Including specific areas for further research helps to acknowledge gaps in knowledge and suggests directions for future studies. You might also suggest potential methodologies or frameworks that could be used in future research to enhance its applicability.

**Author’s response:** Dear Reviewer, thank you very much for your suggestion. We have made the corrections accordingly.

9. The structure is logical, progressing from the background to methods, results, and conclusions. However, can you ensure consistency in terminology, particularly regarding "unimproved drinking water sources," and avoid redundancy? Have you considered defining key terms or abbreviations (e.g., LLR, p-values) for readers who may not be familiar with statistical analyses? In the main text, would it be beneficial to reference recent studies or reports that address similar issues in East Africa to provide context for your findings? If not already included in the full manuscript, would adding visual aids (maps, charts) to effectively illustrate spatial distributions and clusters enhance the reader's understanding? In the discussion section, could you address any limitations of your study, such as potential biases in the data source or challenges encountered during data collection?

**Author’s response:** Dear Reviewer, thank you very much for your comments. We have made the corrections accordingly.

10. Access to drinking water is a fundamental necessity and a human right essential for the dignity and health of everyone. Comment: This statement sets a powerful tone by emphasizing the human right to safe drinking water. Consider adding a citation to reinforce this assertion, as it lends credibility to your claim.

**Author’s response:** Dear Reviewer, thank you very much for your comments. We have made the corrections accordingly.

11. As a basic human right clean, safe drinking water that is readily available year-round, insufficient quantities, uncontaminated by chemicals or dangerous microbes and safe should be available. Comment: The definition of clean, safe drinking water is clear, but the sentence structure is awkward. Consider rephrasing for better clarity: "Clean, safe drinking water, defined as readily available year-round, in sufficient quantities, and free from harmful chemicals or microbes, is a fundamental human right."

**Author’s response:** Dear Reviewer, thank you very much for your comments. We have made the corrections accordingly.

12. According to the World Health Organization and United Nations Children's Fund (WHO/UNICEF) Joint Monitoring Programme, improved drinking-water sources are defined as those that are likely to be protected from external contamination, particularly from fecal matter. Comment: This definition is well-cited and provides essential context. However, consider mentioning the specific year of the report or guidelines to ensure the reader knows the currency of the information.

**Author’s response:** Dear Reviewer, thank you very much for your comments. We have made the corrections accordingly.

13. These sources include household connections, public standpipes, boreholes, protected dug wells, protected springs, and rainwater collection. Comment: This list is informative but could benefit from concise phrasing. Consider using a semicolon to separate items for better readability, or even bullet points for clarity.

**Author’s response:** Dear Reviewer, thank you very much for your comments. We have made the corrections accordingly.

14. In contrast, unimproved water sources encompass unprotected wells, unprotected springs, surface water (e.g., rivers, dams, or lakes), vendor-provided water, bottled water (unless water for other uses is available from an improved source), and tanker truck-provided water. Comment: This contrasts well with the previous sentence. However, the phrase “unless water for other uses is available from an improved source” is cumbersome. Consider simplifying it to enhance readability.

**Author’s response:** Dear Reviewer, thank you very much for your comments. We have made the corrections accordingly.

15. WHO and UNICEF report in 2017 reveals that approximately 3 in 10 people worldwide, or2.1 billion, lack access to safe, readily available water at home. Comment: This statistic is impactful and underscores the significance of the issue. Ensure the citation is formatted consistently to maintain academic rigor.

**Author’s response:** Dear Reviewer, thank you very much for your comments. We have made the corrections accordingly.

16. In addition, around 1 in 4 people lacked safely managed drinking water in their homes in 2020. Comment: This sentence effectively builds on the previous statistic, but it would benefit from a citation for the 2020 data to support your claims.

**Author’s response:** Dear Reviewer, thank you very much for your comments. We have made the corrections accordingly.

17. Half of the world's population that drinks water from contaminated sources is found in Africa, despite the Millennium Development Goals' (MDGs) water target aim being largely successful in various parts of the world. Comment: This is a crucial point that emphasizes the disparity in access to clean water. However, consider rephrasing for clarity: "Despite the MDGs' successes in various regions, Africa still accounts for half of the global population using contaminated water sources."

**Author’s response:** Dear Reviewer, thank you very much for your comments. We have made the corrections accordingly.

18. According to the WHO reports, Sub-Saharan Africa is experiencing the slowest rate of progress in the world. Comment: This statement is strong, but the source should be specified to bolster your argument. Consider providing a citation for the "WHO reports" mentioned here.

**Author’s response:** Dear Reviewer, thank you very much for your comments. We have made the corrections accordingly.

19. Only 54 percent of people used improved drinking water, and only 25 percent in fragile contexts. Comment: While informative, specify what "fragile contexts" refers to for clearer understanding. Are these conflict zones, or economically challenged areas?

**Author’s response:** Dear Reviewer, thank you very much for your comments. We have made the corrections accordingly.

20. A study conducted in 2020 using secondary data estimated that 29% of the 357,979 households in Sub-Saharan Africa used unimproved water for drinking purposes. Comment: This is a significant statistic that supports your argument. However, it would be beneficial to provide a citation for the study to ensure readers can reference it.

**Author’s response:** Dear Reviewer, thank you very much for your comments. We have made the corrections accordingly.

21. In addition, a cross-sectional survey estimated that 38.0% of the population of Sub- Saharan Africa (SSA) reported using an unimproved drinking-water source, although at national levels this was seen to vary between as high as 88.5% in rural Somalia populations to 1.0% in urban populations in Namibia. Comment: The detail on variability among populations is excellent. However, ensure that the statistical data is sourced properly. This information should include a citation for transparency.

**Author’s response:** Dear Reviewer, thank you very much for your comments. We have made the corrections accordingly.

22. In East Africa, the bulk of water at very high risk (68.7%) came from unimproved drinking water sources, including springs and unsecured surface water. Comment: This statistic effectively highlights the risk associated with unimproved water sources. Including a citation would add credibility.

**Author’s response:** Dear Reviewer, thank you very much for your comments. We have made the corrections accordingly.

23. In this study area, the use of unimproved drinking water sources varies from one country to another according to different surveys, with 47% of the population in Ethiopia, 37% in Kenya, 39% in Tanzania, and 41% in Uganda relying on unimproved drinking water sources. Comment: This provides valuable country-specific data but could be better structured. Consider rephrasing for smoother flow: "Surveys indicate variability in the use of unimproved water sources, with 47% of the population in Ethiopia, 37% in Kenya, 39% in Tanzania, and 41% in Uganda relying on such sources."

**Author’s response:** Dear Reviewer, thank you very much for your comments. We have made the corrections accordingly.

24. Climate change, increasing water scarcity, population growth, demographic changes, and urbanization already pose challenges for improved water source supply systems in East Africa. Comment: This sentence outlines critical factors affecting water supply. However, consider using "pose significant challenges" to emphasize the urgency of the issue.

**Author’s response:** Dear Reviewer, thank you very much for your comments. We have made the corrections accordingly.

25. Using unimproved drinking water sources can have significant adverse health effects. Comment: This statement sets up the discussion on health risks well. However, it would be more compelling if it started with "The use of unimproved drinking water sources...".

**Author’s response:** Dear Reviewer, thank you very much for your comments. We have made the corrections accordingly.

26. These include waterborne diseases such as cholera, dysentery, typhoid fever, and hepatitis. Comment: This list is strong and pertinent. Consider explaining how these diseases disproportionately affect vulnerable populations to heighten the stakes of your argument.

**Author’s response:** Dear Reviewer, thank you very much for your comments. We have made the corrections accordingly.

27. Additionally, they can cause diarrheal diseases, which can be severe and life-threatening in children and the elderly. Comment: This adds depth to the health implications. However, try rephrasing to eliminate redundancy with the previous sentence regarding waterborne diseases.

**Author’s response:** Dear Reviewer, thank you very much for your comments. We have made the corrections accordingly.

28. Unimproved water sources can also lead to parasitic infections such as Giardia and Cryptosporidium, causing gastrointestinal issues. Comment: This point effectively diversifies the health risks discussed. Ensure proper citation for the data to maintain academic integrity.

**Author’s response:** Dear Reviewer, thank you very much for your comments. We have made the corrections accordingly.

29. Finally, using unimproved water sources can result in skin infections and other dermatological conditions. Comment: This is a valuable addition to the discussion of health impacts, broadening the scope of adverse effects. Again, cite relevant studies to support this claim.

**Author’s response:** Dear Reviewer, thank you very much for your comments. We have made the corrections accordingly.

30. There was a study conducted in 2024 that determine the pooled prevalence of unimproved drinking water source and its associated factors in East Africa but it didn‟t show the spatial variation of unimproved drinking water sources in the region. Comment: This statement introduces a relevant study but is confusing due to tense and structure. Consider rephrasing for clarity: "A 2024 study assessed the pooled prevalence of unimproved drinking water sources and their associated factors in East Africa but did not address the spatial variation of these sources."

**Author’s response:** Dear Reviewer, thank you very much for your comments. We have made the corrections accordingly.

31. Therefore, conducting spatial analysis of unimproved water sources will help to identify high-risk areas, understand the spatial distribution patterns, and prioritize regions for interventions by establishing a correlation between the locations of unimproved water sources and incidences of waterborne infections. Comment: This statement effectively outlines the study's purpose, but it’s lengthy and could be broken into shorter sentences for better clarity. Consider: "Thus, conducting a spatial analysis of unimproved water sources will identify high-risk areas and help understand spatial distribution patterns. This analysis can prioritize regions for interventions by correlating the locations of unimproved water sources with incidences of waterborne infections."

**Author’s response:** Dear Reviewer, thank you very much for your comments. We have made the corrections accordingly.

32. Therefore, this study aims to determine the spatial distribution of unimproved drinking water sources in East Africa using demographic and health survey (DHS) data from 12 East African countries. Comment: This concluding statement effectively summarizes the aim of the study. Ensure that the importance of this aim is highlighted to create a stronger rationale for the research.

**Author’s response:** Dear Reviewer, thank you very much for your comments. We have made the corrections accordingly.

33. The study was conducted in East Africa. Comment: A clear opening statement. Consider adding a brief description of why East Africa is of interest for this study.

**Author’s response:** Dear Reviewer, thank you very much for your comments. We have made the corrections accordingly.

34. East Africa is a vast and diverse region encompassing the Horn of Africa and the eastern fringes of the Sahara Desert. Comment: Good context. You could enhance this by briefly mentioning the geographical and cultural diversity of the region, which may impact water access and usage.

**Author’s response:** Dear Reviewer, thank you very much for your comments. We have made the corrections accordingly.

35. There are a total of 19 East African countries Comment: Ensure that the focus remains on the 12 countries included in the study to avoid confusion. Consider stating the reasons for including only those specific countries.

**Author’s response:** Dear Reviewer, thank you very much for your comments. We have made the corrections accordingly.

36. A population based crossectional design was conducted. Comment: Specify the time frame of data collection for clarity. Additionally, define what a population-based cross-sectional design entails for readers who may not be familiar with the terminology.

**Author’s response:** Dear Reviewer, thank you very much for your comments. We have made the corrections accordingly.

37. Data for this study were derived from the most recent standard Demographic and Health Surveys (DHS) of the 12 East African countries. Comment: Good justification for data selection. Mention any criteria for choosing the most recent surveys.

**Author’s response:** Dear Reviewer, thank you very much for your comments. We have made the corrections accordingly.

38. The DHS is a nationally representative survey that provides data for monitoring indicators of population dynamics, nutrition, and health with large sample size. Comment: Consider citing specific indicators relevant to your study to provide clarity on the data's utility.

**Author’s response:** Dear Reviewer, thank you very much for your comments. We have made the corrections accordingly.

39. The permission to use the data was granted from DHS program (S1File). Comment: Clarify what S1File is, as it might not be obvious to all readers.

**Author’s response:** Dear Reviewer, thank you very much for your comments. We have made the corrections accordingly.

40. Study Population and Sampling “DHS used a multistage (two stage) cluster sampling technique to recruit the samples using EAs or (Enumeration Areas) or clusters and households as primary and secondary sampling units, respectively.” Comment: This is well-stated. You might briefly explain why a multistage sampling technique was chosen and its advantages for this type of study.

**Author’s response:** Dear Reviewer, thank you very much for your comments. We have made the corrections accordingly.

41. A total of 206,748 households were sampled in 12 East African countries and 8040 clusters was considered for analysis. Comment: Excellent detail on sample size. You may want to note any relevant demographic information about the households sampled (e.g., urban vs. rural).

**Author’s response:** Dear Reviewer, thank you very much for your comments. Since, the study was a spatial analysis we didn’t intend to include further descriptive results beside the included descriptions.

42. In DHS there is a probability that some of the regions or countries‟ were oversampled and some were under-sampled specifically in large countries. Comment: Acknowledging potential sampling biases is crucial. Consider discussing how these biases might affect the study's findings.

**Author’s response:** Dear Reviewer, thank you very much for your comments. We have made the corrections accordingly.

43. Data Presentation: The results are presented clearly, but the addition of visual elements such as maps and graphs could significantly enhance comprehension. Visual aids will help convey the spatial distribution of unimproved water sources more effectively.

**Author’s response:** Dear Reviewer, thank you very much for your suggestion. However, all the figures included in the manuscript, could significantly enhance the presentation and effectively convey the spatial distribution of unimproved water sources.

44. Contextual Background: While the background section provides a solid foundation, incorporating more context regarding the socio-economic implications of unimproved water sources would deepen the understanding of the research's significance.

**Author’s response:** Dear Reviewer, thank you very much for your suggestion. We have made the corrections accordingly.

45. Limitations: The limitations section is somewhat brief. A more comprehensive discussion on how the study's generalizability might be affected by excluding certain remote or conflict-affected areas would provide a more balanced perspective. Addressing potential biases in the data or challenges faced during the analysis is crucial for transparency.

**Author’s response:** Dear Reviewer, thank you very much for your suggestion. We have made the corrections accordingly.

46. Future Research Suggestions: I recommend including suggestions for future research, such as exploring the effectiveness of specific interventions or examining the long-term health impacts of unimproved water sources. This could guide subsequent studies in this important area.

**Author’s response:** Dear Reviewer, thank you very much for your suggestion. We have made the corrections accordingly.

47. Ethical Considerations: I have no concerns regarding dual publication or research ethics. The research appears to adhere to ethical guidelines, and the authors have adequately addressed the use of data from demographic and health surveys.

**Author’s response:** Dear Reviewer, thank you very much.

48. Conclusion and Recommendations: The recommendations provided are actionable and address the critical need for collaboration among stakeholders. However, highlighting successful case studies or ongoing efforts within East Africa that align with these recommendations would offer a practical perspective and underscore the feasibility of proposed actions. In summary, while the manuscript is well-structured and addresses an important public health challenge, it would benefit from refinements in methodological clarity, contextual background, and data presentation. I believe that addressing these points will enhance the manuscript's impact on the understanding and management of unimproved drinking water sources in East Africa.

**Author’s response:** Dear Reviewer, thank you very much for your suggestion. We have made the corrections accordingly.

49. Policy and Practice Implications the recommendations are practical and targeted, such as prioritizing hotspot regions for infrastructure improvements and building climate resilience. The call for collaboration with organizations like WHO, USAID, and development banks aligns with the complex, cross-border challenges of water access in East Africa. However, including examples of successful partnerships in similar contexts could clarify potential outcomes. Additionally, discussing possible barriers like funding constraints or political instability and suggesting strategies to address them would add depth to the discussion.

**Author’s response:** Dear Reviewer, thank you very much for your suggestion. We have made the corrections accordingly.

**Comments from Reviewer #6:**

1. The study's goal is to figure out the spatial distribution of unimproved drinking water sources in East Africa. This study found a significant spatial clusters, hotspots, and outliers of unimproved drinking water sources in several East African countries. The study identified significant spatial clusters, hotspots, and outliers of unimproved drinking water sources in East Africa, and recommends that these areas be prioritized for expanding water treatment facilities, improving water distribution, and protecting water sources, with collaboration from East African governments, international organizations, and other stakeholders. Spatial clusters of unimproved drinking water sources were detected throughout East Africa, indicating a major global clustering tendency. A primary cluster covering nearly all of Madagascar and parts of Mozambique's coastal regions was identified, exhibiting a high Log Likelihood Ratio (LLR) of 5221.25 and a p-value < 0.000. Secondary clusters were found across all 12 countries included in the analysis, with LLRs ranging from 11.93 to 3135.38 and p-values < 0.05.

**Author’s response:** Dear Reviewer, thank you very much understanding.

2. Some portions, particularly the introduction and methodology, might be condensed to improve readability. Breaking down long sentences and reducing difficult phrases would make the text more understandable. Ensure consistency when referring to tools and analysis. For example, utilizing consistent language for software versions and statistical tests promotes clarity.

**Author’s response:** Dear Reviewer, thank you very much for your suggestion. We have made the corrections accordingly.

3. While the methods are thorough, including a simple summary table or flowchart could help readers grasp the analysis workflow, particularly those unfamiliar with spatial statistics.

**Author’s response:** Dear Reviewer, thank you very much for your suggestion. We have made the corrections accordingly.

4. Including brief comparisons or context from similar research in other locations may help to highlight the specific elements of East Africa's difficulties and deepen the conversation.

**Author’s response:** Dear Reviewer, thank you very much for your suggestion. We have made the corrections accordingly.

5. Clarifying which identified hotspots should be prioritized based on severity or population impact could make the recommendations more actionable.

**Author’s response:** Dear Reviewer, thank you very much for your suggestion. We have made the corrections accordingly.

6. The suggestions for future research could be expanded to include potential methods or additional data sources that could refine or complement the current findings.

**Author’s response:** Dear Reviewer, thank you very much for your suggestion. We have made the corrections accordingly.
