# Supplementary material for: Spatial analysis of unimproved drinking water source in East Africa: Using Demographic and Health Survey (DHS) data from 2012–2023
Source: PLoS One. 2025 Mar 20;20(3):e0318189. doi: 10.1371/journal.pone.0318189 (PMC11925304; doi:10.1371/journal.pone.0318189)
Supplement: Attachment — Submitted filename: Response_to_Reviewers_auresp_2.docx [file pone.0318189.s008.docx]

**Responses to the Editors and review’s comments**

**Dear PLOS ONE editorial team,**

Thank you for giving us the opportunity to submit a revised draft of the manuscript, and we would also like to thank you for your crucial comments on our paper (Manuscript ID: **PONE-D-24-33669** **R1**). We are very concerned and have combined all the suggested comments provided, which we believe strengthen our paper, and we hope this will render our paper eligible for consideration for publication in your reputed journal. We appreciate the time and effort that you and the reviewers dedicated to providing feedback on our manuscript and are grateful for the insightful comments and valuable improvements to our paper for publication.

The authors would like to inform you that we have addressed the comments and recommendations of the handling editor point by point. In addition, throughout our revision, we made our best corrections too. All changes made to the original version are highlighted using tracking changes and attached as **“Revised Manuscript with Track Changes”**. The unmarked copy of the manuscript is also attached as **“Manuscript”**. In addition, please see below a rebuttal letter that responds to each point raised by the handling editor, and this letter is also attached to the submission as **“Response to Reviewers”**.

**Response to editor’s comments**

**Comments from the handling editor:**

1. Please review your reference list to ensure that it is complete and correct. If you have cited papers that have been retracted, please include the rationale for doing so in the manuscript text, or remove these references and replace them with relevant current references. Any changes to the reference list should be mentioned in the rebuttal letter that accompanies your revised manuscript. If you need to cite a retracted article, indicate the article’s retracted status in the References list and also include a citation and full reference for the retraction notice.

**Author’s response:** Dear Editor, Thank you for your guidance regarding the reference list. We have carefully reviewed all the references cited in our manuscript to ensure their accuracy and relevance. We confirm that none of the cited references have been retracted.

2. There are still a few corrections needed. Please pay attention and make the necessary changes to your manuscript.

**Author’s response:** Dear Editor, thank you very much for your valuable recommendations. We have carefully considered your suggestions and made the necessary corrections accordingly.

**Comments from Reviewer #2:**

1. I have carefully assessed the manuscript and provided detailed feedback to address the points requiring clarification and improvement. Overall, I found the study was well-conducted and addressed my comments. I have seen all areas, where further refinement could enhance the manuscript’s clarity and impact. Please find my review attached to this email for your reference. I hope my comments contribute to improving the quality of the manuscript. Should you require any additional clarification or further input from me, please feel free to contact me.

**Author’s response:** Dear reviewer, thank you very much for your insightful questions, valuable suggestions, and thoughtful recommendations. Your feedback has significantly enhanced the overall quality of the manuscript.

2. The first letter of each keyword must be a Capital letter.

**Author’s response:** Dear reviewer, thank you very much for your comment. We have made corrections accordingly.

3. Data from 12 East African countries. Please list the countries included in your study.

**Author’s response:** Dear reviewer, thank you very much for your comment. We have made corrections accordingly.

4. A total of 206,748 households were sampled in 12 East African countries… Did you use sampling weight?

**Author’s response:** Dear reviewer, thank you very much for your question. Yes, we used sampling weights, and we have already provided a detailed explanation in lines 147 to 151.

5. Data management and analysis were conducted using Microsoft Excel 2010, STATA 17, ArcGIS software version 10.7, and Kuldorff’s SaTScan 10.1. Excel was used for initial data cleaning, STATA for statistical weighting and data merging, ArcGIS software version 10.7 for geospatial analysis and mapping, and Kuldorff’s SaTScan 10.1 for identifying spatial clusters. There is no need to list the software you have used here; you can list the software you have used for data analysis in the methods and material section. Simply list the methods you have used for data analysis here.

**Author’s response:** Dear reviewer, thank you very much for your comment. We have made corrections accordingly.

6. Your conclusion is vast, please give your conclusion based on your result.

**Author’s response:** Dear reviewer, thank you very much for your comment. We have made the necessary corrections. The majority of the conclusions section was recommendations, which led to its length. We have minimized it as much as possible while ensuring that the feedback from other reviewers is not compromised.

**Comments from Reviewer #4:**

1. Miner revision of the manuscript Spatial analysis of unimproved drinking water source in East Africa: Using Demographic and Health Survey (DHS) data from 2012-2023

**Author’s response:** Dear reviewer, thank you very much for your effort to improve the overall quality of the manuscript.
